# A Robust Hypoxia Risk Score Predicts the Clinical Outcomes and Tumor Microenvironment Immune Characters in Bladder Cancer

**DOI:** 10.3389/fimmu.2021.725223

**Published:** 2021-08-13

**Authors:** Zhi Liu, Qiao Tang, Tiezheng Qi, Belaydi Othmane, Zhe Yang, Jinbo Chen, Jiao Hu, Xiongbing Zu

**Affiliations:** ^1^Department of Urology, Xiangya Hospital, Central South University, Changsha, China; ^2^Department of Urology, The Second Affiliated Hospital, Guizhou Medical University, Kaili, China; ^3^Xiangya School of Medicine, Central South University, Changsha, China; ^4^Department of Histology and Embryology, School of Basic Medicine, Guizhou Medical University, Guiyang, China

**Keywords:** bladder cancer, tumor microenvironment, hypoxia, immunotherapy, chemotherapy, risk score

## Abstract

**Background:**

Bladder cancer (BLCA) is one of the most common urinary malignancies with poor prognosis. There is an unmet need to develop novel robust tools to predict prognosis and treatment efficacy for BLCA.

**Methods:**

The hypoxia-related genes were collected from the Molecular Signatures Database. The TCGA-BLCA cohort was downloaded from the Cancer Genome Atlas and then was randomly divided into training and internal validation sets. Two external validation cohorts were gathered from Gene Expression Omnibus. Also, another independent validation cohort (Xiangya cohort) was collected from our hospital. The Cox regression model with the LASSO algorithm was applied to develop the hypoxia risk score. Then, we correlated the hypoxia risk score with the clinical outcomes, the tumor microenvironment (TME) immune characteristics, and the efficacy prediction for several treatments, which included cancer immunotherapy, chemotherapy, radiotherapy, and targeted therapies.

**Results:**

Hypoxia risk score was an independent prognostic factor. A high-risk score indicated an inflamed TME based on the evidence that hypoxia risk score positively correlated with the activities of several cancer immunity cycles and the infiltration levels of many tumor-infiltrating immune cells, such as CD8 + T cells, Dendritic cells, and NK cells. Consistently, the hypoxia risk score was positively related to the expression of several immune checkpoints, such as PD-L1, PD-1, CTLA-4, and LAG-3, as well as the T cell inflamed score. Furthermore, the hypoxia risk score positively correlated with the enrichment scores of most immunotherapy-positive gene signatures. Therefore, patients with higher risk score may be more sensitive to cancer immunotherapy. Meanwhile, the hypoxia risk score was positively related to the sensitivities of several chemotherapeutic drugs, including Cisplatin, Docetaxel, Paclitaxel, Bleomycin, Camptothecin, and Vinblastine. Similarly, the enrichment scores for radiotherapy-predicted pathways and EGFR ligands were higher in the high-risk score group. Conversely, the enrichment scores of several immunosuppressive oncogenic pathways were significantly higher in the low-risk score group, such as the WNT-β-catenin network, PPARG network, and FGFR3 network.

**Conclusions:**

We developed and validated a new hypoxia risk score, which could predict the clinical outcomes and the TME immune characteristics of BLCA. In general, the hypoxia risk score may aid in the precision medicine for BLCA.

## Introduction

Bladder cancer (BLCA) is one of the most common urinary malignancies (1). The prognosis of non-muscle invasive BLCA is good, but once the muscle-invasive stage or locally metastatic progression is reached, the prognosis is deemed to be poor (2). There are various treatment options such as neoadjuvant or adjuvant chemotherapy, immunotherapy, and several targeted therapies (2). Nonetheless, the overall response rate of these treatments was low, which was caused by many primary or secondary resistance mechanisms, such as hypoxia (3). In addition, BLCA was a heterogeneous disease, which impedes the achievement of precision medicine (4). Therefore, it is challenging to develop novel robust tools to predict prognosis and treatment efficacy.

Hypoxia is a hallmark of the tumor microenvironment (TME) and plays critical roles in cancer initiation, progression, and treatment resistance in various cancers, including BLCA (3, 5–7). With the popularization of transcriptome sequencing technology, many hypoxia-related risk signatures have been developed in multiple tumors to predict prognosis and treatment efficacy (8–11). As for BLCA, Yang et al. developed and validated a hypoxia gene signature that could predict survival and identify those patients likely to benefit from the addition of carbogen and nicotinamide to radiotherapy (12). Nowadays, immune checkpoint blockade (ICB), such as anti-PD-L1/PD-1 therapy, has revolutionized the treatments for advanced cancers. More and more clinical trials highlighted the roles of ICB in first-line or second-line treatments for advanced BLCA (13–15). The ICB response rate is closely related to the status of the tumor immune microenvironment (16, 17). However, there is no research to systematically correlate hypoxia-related signature with the tumor immune microenvironment in BLCA.

In this study, we integrated multiple independent BLCA data sets to develop a new hypoxia risk score and correlate it with the clinical outcomes, the TME characteristics, and the treatment efficacy prediction.

## Materials and Methods

### Data Retrieval and Preprocessing

#### External Public Cohorts

We downloaded the mRNA expression matrix (FPKM) of 414 BLCA tumor samples and 19 normal tissues from the Cancer Genome Atlas (TCGA) (https://portal.gdc.cancer.gov/). Then, the FPKM value was converted into TPM value. Two external validation GSE cohorts with detailed survival data were gathered from Gene Expression Omnibus (GEO), namely GSE32894 and GSE13507. GSE32894 (platform: GPL6947) included 224 BLCA samples and GSE13507 (platform: GPL6102) included 165 BLCA samples. 200 hypoxia-related genes were collected from the Molecular Signatures Database (MSigDB) (http://www.gsea-msigdb.org/gsea/msigdb/cards/HALLMARK_HYPOXIA.html).

#### Xiangya Cohort

As reported in our previous study, 57 BLCA samples were sequenced on a BGISEQ-500 platform (BGI-Shenzhen, China) (13). Among these patients, 56 patients were successfully followed up.

The detailed information of these cohorts was provided in **Supplementary Table 1**.

### Identification of Differentially Expressed Hypoxia Genes (Hypoxia DEGs) and Functional Analysis

We used the empirical Bayesian approach of the limma R package to identify hypoxia DEGs between bladder cancer samples and normal samples. The criteria used to determine hypoxia DEGs were set with the |log(fold change)|>1 and the adjusted P-value < 0.05 (18). Then, we performed Gene Ontology (GO) and Kyoto Encyclopedia of Genes and Genomes (KEGG) analyses based on those hypoxia DEGs by using the ClusterProfiler R package (19). In addition, we explored the protein–protein interaction (PPI) network of those hypoxia DEGs by using the String database and Cytoscape software.

### Development and Validation of the Hypoxia Risk Score

First, we performed univariate Cox analysis to screen the prognostic hypoxia DEGs in the TCGA cohort. Then, the TCGA-BLCA cohort was randomly divided into training and validation sets with a ratio of 4:1. In the TCGA-BLCA training set, the least absolute shrinkage and selector operation (LASSO) algorithm was further applied to identify the optimal candidate hypoxia DEGs with the best discriminative capability. Finally, the hypoxia risk score was developed based on those optimal candidate hypoxia DEGs, weighted using the LASSO coefficient as follows:

Risk score=Σ βi∗RNAi,where βi is the coefficient of the i−th.

Patients were classified into high and low hypoxia risk score groups based on the median of the risk score. The Kaplan–Meier method was applied to plot the survival curves. The log-rank test was used to statistically compare the groups in order to estimate the prognostic significance of the hypoxia risk score. The statistical performance for predicting survival of the hypoxia risk score was calculated using the tROC R package. We further validated the role of the hypoxia risk score algorithm in the TCGA validation set, GSE32894, GSE13507, and Xiangya cohort. We then correlated the hypoxia risk score with the tumor grade and stage. The remarkable thing was that there were only 19 patients who were diagnosed with a high grade in the TCGA-BLCA cohort. Therefore, the uni- and multivariate Cox analyses were performed to identify independent prognostic factors based on age, gender, tumor stage, and hypoxia risk score. Meanwhile, we developed a systematic nomogram based on these important clinicopathological characters and the hypoxia risk score. The statistical performance of the nomogram was validated by using clinical decision curves.

### Depicting the Hallmarks of Molecular Subtypes and TME of BLCA

As reported in our previous study, seven independent molecular subtype systems were analyzed, such as the UNC, TCGA, and Consensus systems (13). Twelve molecular subtype-specific signatures were collected and correlated with the hypoxia risk score. Several immunological characteristics of TME and the corresponding algorithms were also described in our previous study (13). To briefly summarize, we calculated the activities of the cancer immunity cycles, such as cancer antigen release and presentation, immune cell trafficking, and killing of cancer cells. Then we estimated the infiltration levels of several tumor-infiltrating immune cells (TIICs) by using six independent algorithms, such as Cibersort-ABS, TIMER, and TIP.

### Gene Set Variation Analysis (GSVA) and Efficacy Prediction of Several Treatments

GSVA is a non-parametric and unsupervised method that is commonly used to estimate the difference in the activity of pathways or biological processes in the samples of an expression dataset (20). To investigate the difference in 50 hallmark pathways between hypoxia risk score groups, we performed GSVA enrichment analysis using the “GSVA” R packages. The corresponding pathways were collected from the MSigDB (21). We predicted the chemotherapeutic response of individuals by using the pRRophetic package based on the data from Genomics of Drug Sensitivity in Cancer (GDSC) (https://www.cancerrxgene.org/) (22). We calculated the IC50 value of six common chemotherapeutic drugs, including Cisplatin, Docetaxel, Paclitaxel, Bleomycin, Camptothecin, and Vinblastine. In addition, we collected several potential predictors for the efficacy of ICB, such as 20 inhibitory immune checkpoints, the pan-cancer T cell inflamed score (TIS), and 19 gene signatures positively correlated with the clinical response of immunotherapy in BLCA (13). Finally, several signatures related to the clinical response of radiotherapy and targeted therapies were also collected. The ssGSEA algorithm was applied to calculate the enrichment scores of these signatures.

### Statistical Analysis

We analyzed the correlations between variables using Pearson or Spearman coefficients. We compared the difference in continuous variables between binary groups using a t-test or Mann-Whitney U test. The empirical Bayesian approach of the limma R package was used to identify hypoxia DEGs. The LASSO algorithm was applied to identify the optimal hypoxia DEGs candidates with the best discriminative capability. The Kaplan-Meier method was applied to plot the survival curves, while the log-rank test was applied to calculate statistical significance. The receiver-operating characteristic (ROC) curves were plotted to calculate the accuracy of the hypoxia risk score in predicting the survival and molecular subtypes. Statistical tests were two-sided, and the level of significance was set at *P* < 0.05. All statistical data analyses were implemented using R software.

## Results

### Functional Analysis of Hypoxia DEGs

A total of 94 hypoxia DEGs were screened between BLCA and normal tissues. Among them, 55 hypoxia genes were highly expressed in BLCA, while 39 hypoxia genes were down-expressed (**Supplementary Table 2** and **Supplementary Figures 1A, B**). **Supplementary Figure 1A** showed the top 20 hypoxia DEGs. Results of GO analysis indicated that these hypoxia DEGs were enriched in several pathways, including monosaccharide metabolic process, lysosomal lumen, monosaccharide binding, and carbohydrate kinase activity **(** **Supplementary Figure 1C**). Results of KEGG analysis showed that these hypoxia DEGs were enriched in Glycolysis/Gluconeogenesis, Starch and sucrose metabolism, and the Pentose phosphate pathway (**Supplementary Figure 1D**). Notably, the most common enrichment pathways were glucose metabolism-related pathways. **Supplementary Figure 1E** showed the PPI network of these hypoxia DEGs, which suggested that these hypoxia DEGs were closely correlated with each other.

### Development and Internal Validation of a Hypoxia Risk Score in the TCGA-BLCA Cohort

**Figure 1:**
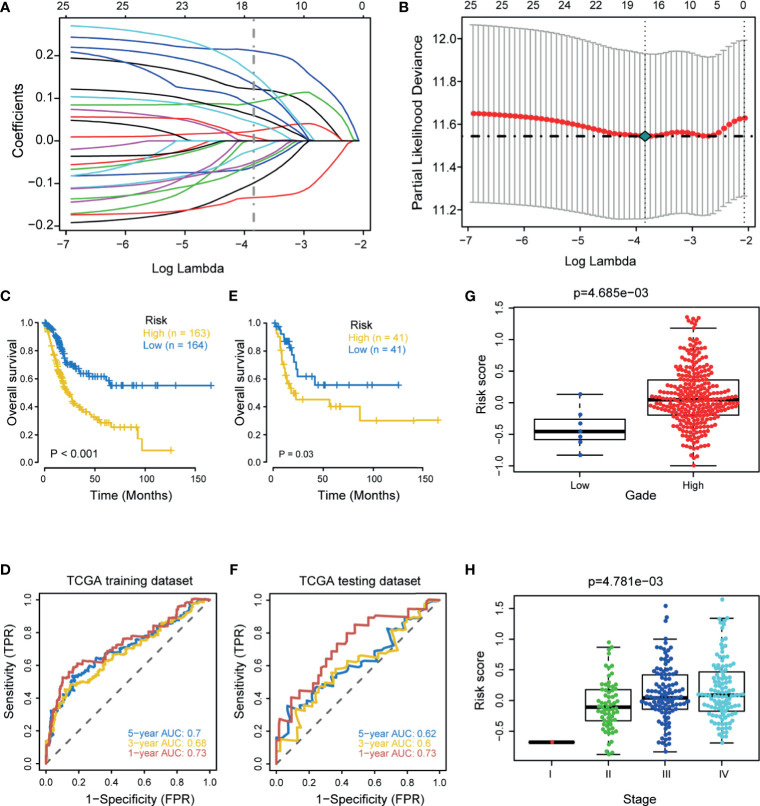
Development and internal validation of a hypoxia risk score in the TCGA-BLCA cohort. **(A)** LASSO coefficient profiles of 25 prognostic hypoxia genes in TCGA training cohort. The coefficient profile plot was developed against the log (Lambda) sequence. **(B)** Cross-validation for turning parameter selection *via* minimum criteria in the LASSO regression model. Two dotted vertical lines were plotted at the optimal values using the minimum criteria. Optimal RNAs with the best discriminative capability (16 in number) were selected for developing the hypoxia risk score. **(C, D)** Development of the hypoxia risk score in TCGA training set and the predictive accuracy of the hypoxia risk score for survival. **(E, F)** Validation of the hypoxia risk score in TCGA validation set. **(G, H)** Associations between the hypoxia risk score and tumor grade and stage.

First, we performed univariate Cox regression analysis based on these hypoxia DEGs in the TCGA-BLCA cohort. Next, we screened 25 prognostic hypoxia genes, including HDLBP, SLC2A3, SRPX, GALK1, HEXA, PAM, ANKZF1, CASP6, ANXA2, AKAP12, VEGFA, XPNPEP1, DCN, BGN, KDELR3, SDC4, TPI1, TGFB3, STC1, WISP2, CCNG2, GAPDH, SLC2A1, HS3ST1, and VHL. Then, we further identified 16 optimal candidates with minimal lambda (0.0214) to generate the hypoxia risk score by using the LASSO algorithm in the TCGA training cohort (**Figures 1A, B** **)**. The coefficients of these 16 genes were shown in **Supplementary Table 3**. In the TCGA training cohort, patients were classified into low and high risk score groups. Notably, Patients in the high risk score group had poorer overall survival (OS) when compared with patients in the low risk score group (**Figure 1C**). The accuracy of the hypoxia risk score in predicting 1-, 3-, and 5-year OS were 0.73, 0.68, and 0.70, respectively (**Figure 1D**). More importantly, we successfully validated the role of the hypoxia risk score in predicting OS in the TCGA validation cohort (**Figures 1E, F** **)**. In addition, we performed subgroup analyses based on stage, grade, gender, and age. As expected, the high-risk score group predicted a worse prognosis in almost all subgroups (**Supplementary Figure 2**). However, the hypoxia risk score was not a significant prognosis predictor in the low-grade subgroup; this might be due to the small sample size.

### Associations Between the Hypoxia Risk Score and Clinicopathological Characters

**Figure 2:**
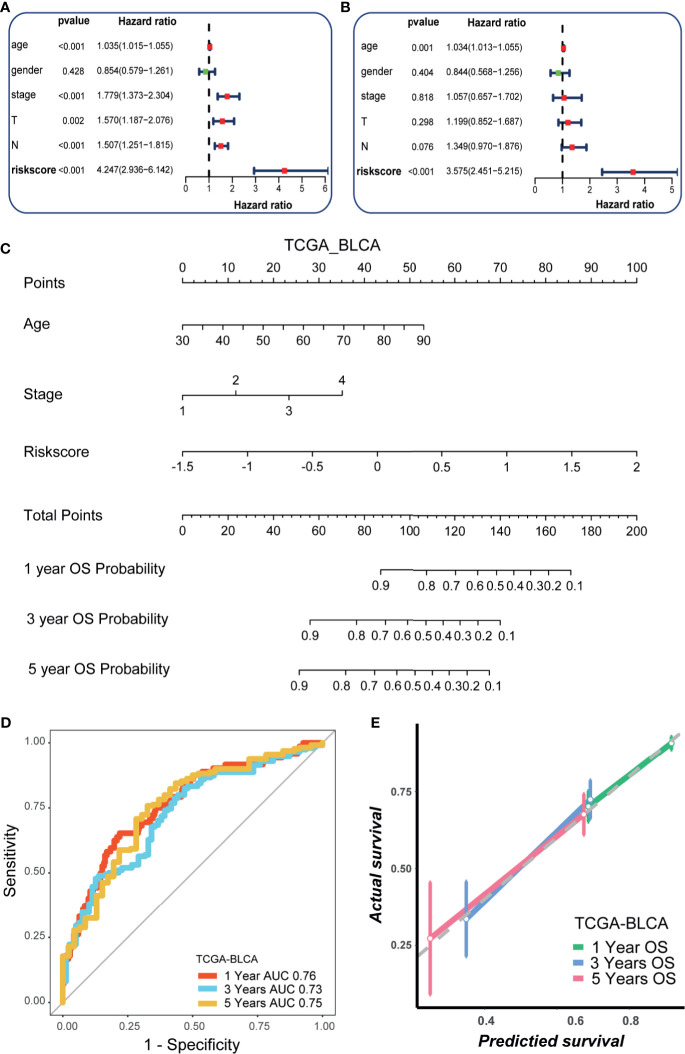
Development of a nomogram by integrating the hypoxia risk score and clinicopathological characters in TCGA-BLCA cohort. **(A)** Results of univariate Cox analysis. **(B)** Results of multivariate Cox analysis. **(C)** The developed nomogram to predict the 1-year, 3-year, and 5-year overall survival. **(D)** The ROC curves of the nomogram. **(E)** Calibration curves of the nomogram measured by Hosmer-Lemeshow test.

In line with the prognostic value of the hypoxia risk score, the risk score was significantly higher in patients with higher grade and stage (**Figures 1G, H** **)**. We further validated these results in three external cohorts (**Supplementary Figure 3**). Then, we performed univariate Cox analysis and revealed that the stage and hypoxia risk score were significant prognostic factors (**Figure 2A**). Further multivariate Cox analysis reconfirmed that the hypoxia risk score was an independent prognostic factor (**Figure 2B**). These results highlighted that the hypoxia risk score may be a promising predictive marker for the prognosis of BLCA patients. In order to promote the clinical application of the hypoxia risk score, we developed a comprehensive nomogram by integrating the hypoxia risk score and several critical clinicopathological characters, such as age and the tumor stage (**Figure 2C**). Although the tumor stage was not an independent prognostic factor in multivariate Cox analysis, its clinical value for patients with BLCA was significant. Therefore, we included the stage in the final nomogram. The predictive accuracy of the nomogram for 1-, 3-, and 5-year OS were 0.76, 0.73, and 0.75, respectively (**Figure 2D**). As shown in the calibration curves (**Figure 2E**), the nomogram-predicted OS was highly consistent with the actual OS, highlighting the clinical significance of this integrated nomogram.

### External Validation of the Hypoxia Risk Score and the Nomogram

**Figure 3:**
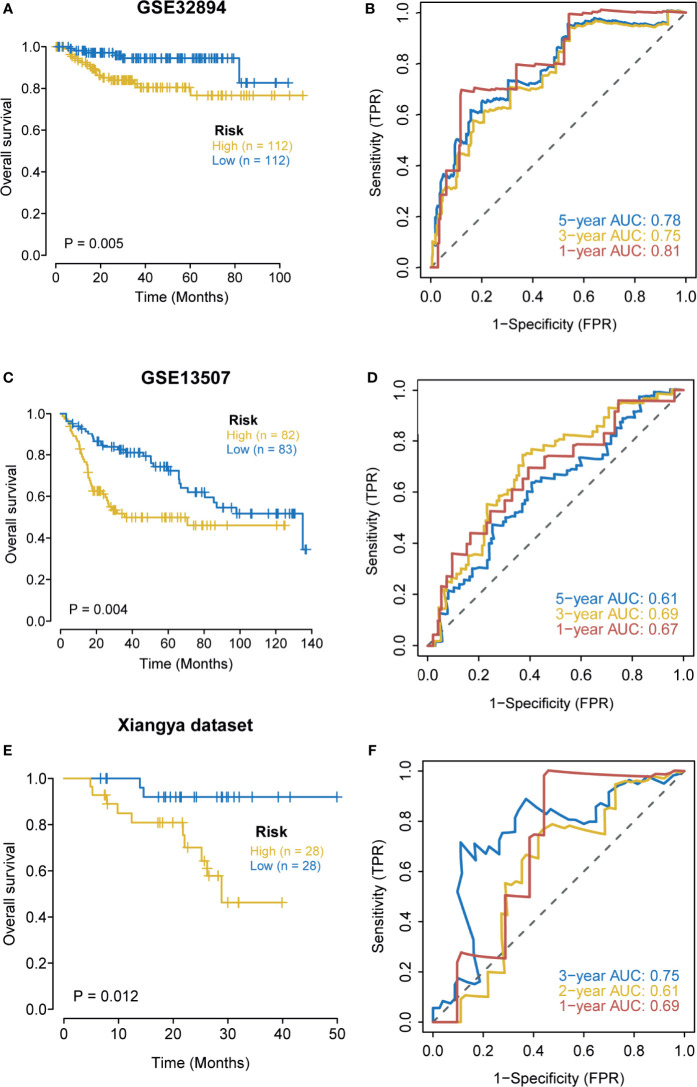
External validation of the hypoxia risk score. **(A, B)** Validation of the hypoxia risk score in GSE32894. **(C, D)** Validation of the hypoxia risk score in GSE13507. **(E, F)** Validation of the hypoxia risk score in Xiangya cohort.

We further explored the predictive value of the hypoxia risk score for prognosis in the GSE32894, GSE13507, and Xiangya cohorts. As expected, in the GSE32894 cohort, patients in the high-risk score group had poorer OS when compared with patients in the low-risk score group (**Figure 3A**). The predictive accuracy for 1-, 3-, and 5-year OS were 0.81, 0.75, and 0.78, respectively (**Figure 3B**). Similarly, in GSE13507, a higher risk score predicted a poorer OS (**Figure 3C**). The predictive accuracy for 1-, 3-, and 5-year OS were 0.67, 0.69, and 0.61, respectively (**Figure 3D**). In the Xiangya cohort, a higher risk score also predicted a poorer OS (**Figure 3E**). The predictive accuracy for 1-, 2-, and 3-year OS were 0.69, 0.61, and 0.75, respectively (**Figure 3F**). Despite the risk score being a prognostic factor in univariate analysis, it was not an independent prognosis predictor in multivariate analysis (**Supplementary Figure 4**). The small sample size and the different patient compositions may cause this phenomenon in these external cohorts. We further explored the role of the nomogram in these external cohorts. As shown in **Supplementary Figures 5A, C, E**, the nomogram predicted the OS with high accuracy (all AUCs were more than 0.8) in three external cohorts, especially in the Xiangya cohort where the predictive accuracy for 3-year OS reached 0.97 (**Supplementary Figure 5 E**). Meanwhile, the calibration curves indicated that the nomogram-predicted OS was highly consistent with the actual OS (**Supplementary Figures 5B, D, F**).

### Hypoxia Risk Score Correlated With Immune Characters of TME and Predicted the Clinical Response of ICB

**Figure 4:**
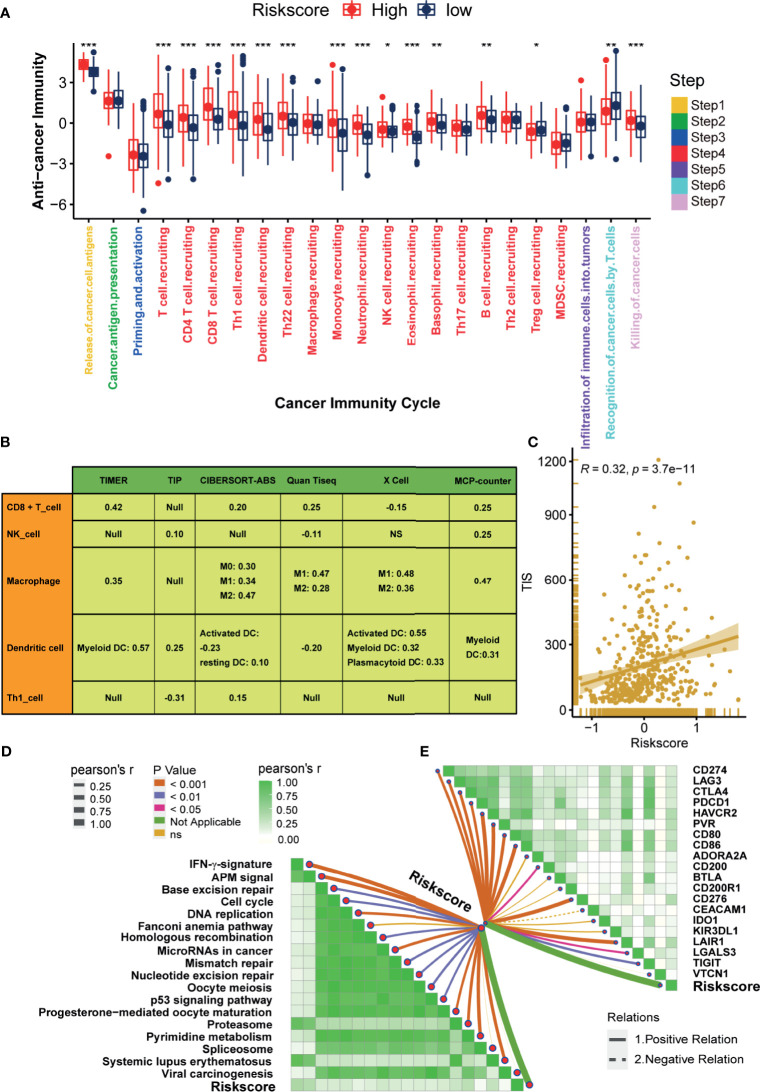
Hypoxia risk score correlated with immune characters of TME and predicted the clinical response of ICB. **(A)** Differences in activities of the cancer immunity cycles between high- and low-risk score groups. **(B)** The correlations between the hypoxia risk score and several immune cells, including CD8 + T cells, NK cells, Macrophage, Dendritic cells, and TH1 cells. **(C)** The correlations between the hypoxia risk score and T cell inflamed score (TIS). **(D)** The correlations between hypoxia risk score and the enrichment scores of immunotherapy-predicted pathways. **(E)** The correlations between hypoxia risk score and immune checkpoints. (*P < 0.05; **P < 0.01; ***P < 0.001).

The state of the tumor immune microenvironment determines the fate of cancer cells and the efficacy of immunotherapy. We first analyzed the correlations between the risk score and the activities of cancer immunity cycles. Obviously, the activities of several anti-cancer immune responses, such as the release of cancer cell antigens, T cell recruiting, CD8 T cell recruiting, Th1 cell recruiting, NK cell recruiting, and killing of cancer cells, were significantly higher in the high-risk score group (**Figure 4A**). As a result, the infiltration levels of the corresponding TIICs, such as CD8 T cells, NK cells, Th1 cells, and Dendritic cells, were positively related to the hypoxia risk score (**Figure 4B**). Based on these data, we proposed that a high-risk score may indicate an inflamed phenotype that may be more sensitive to ICB. Therefore, we subsequently correlated the risk score with several predictors of ICB efficacy. The risk score was positively related to the TIS (**Figure 4C**). Meanwhile, the risk score was also positively related to the expression of many immune checkpoints (such as CD274, CTLA4, and PDCD1) and the enrichment scores of immunotherapy response-related gene signatures (**Figures 4D, E** **)**. Furthermore, we performed subgroup analyses to validate the robustness of the hypoxia risk score in predicting the TME immune characters. As shown in **Supplementary Figures 6** **–** **11**, all the results suggested that the hypoxia risk score may be a potential predictor of ICB efficacy in BLCA.

### Hypoxia Risk Score Stratified the Molecular Subtypes and Aided in Precision Medicine

**Figure 5:**
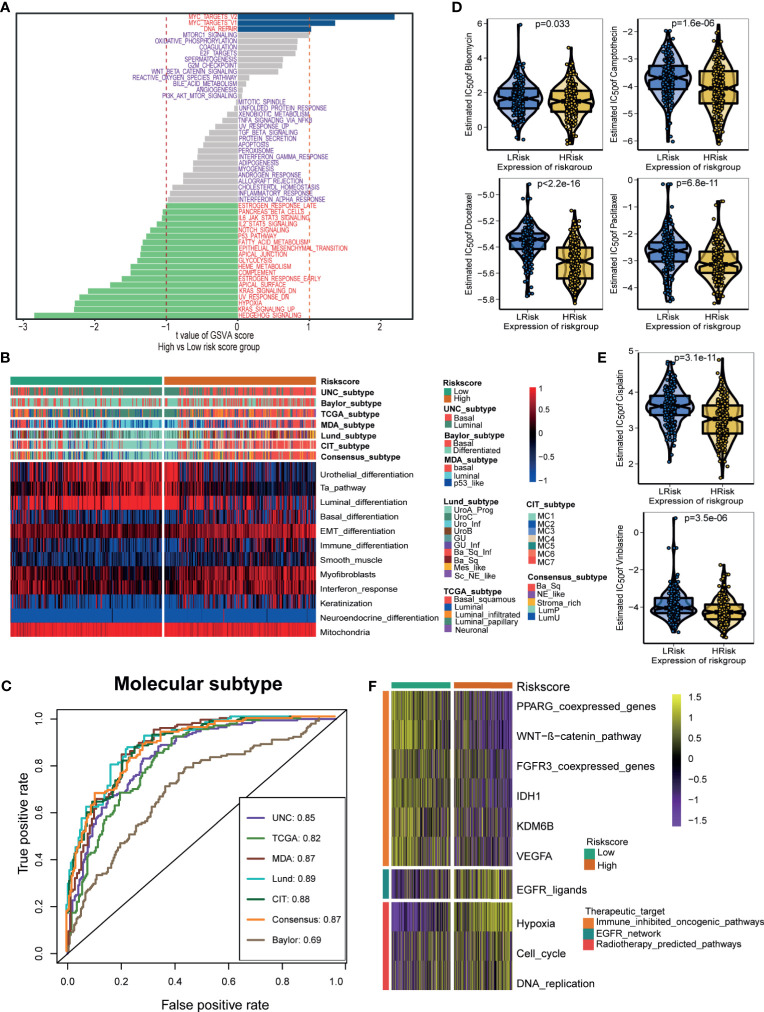
Hypoxia risk score stratified the molecular subtypes and aided in precision medicine. **(A)** The difference on the hallmark gene sets between hypoxia risk score groups. **(B)** The associations between the hypoxia risk score groups and the molecular subtypes in seven different algorithms. **(C)** The predictive accuracy of hypoxia risk score for molecular subtypes in seven different algorithms. **(D, E)** The difference on the therapeutic sensitivities of six chemotherapy drugs, including Cisplatin, Docetaxel, Paclitaxel, Bleomycin, Camptothecin, and Vinblastine. **(F)** Correlations between hypoxia risk score and the enrichment scores of several therapeutic signatures such as targeted therapies and radiotherapy.

Differences in the enrichment scores of 50 hallmark signaling pathways suggested significantly distinct biological functions between the hypoxia risk score groups **(** **Figure 5A**). Myc targets signaling and DNA repair signaling were the top enriched signatures in the low-risk score group. In contrast, Hedgehog signaling, KRAS signaling, and hypoxia signaling were the top enriched signatures in the high-risk score group. These results indicated that the hypoxia genes may influence the progression of BLCA by regulating these hallmark pathways. **Figure 5B** displayed the correlations between the hypoxia risk score and seven classical molecular subtype classifications. Notably, the high-risk score group indicated the basal subtype, which was characterized by basal differentiation, EMT differentiation, myofibroblasts, immune differentiation, and interferon response. In contrast, the low-risk score group suggested the luminal subtype, which was characterized by luminal differentiation and the Ta pathway. In general, the hypoxia risk score could predict, with high accuracy, the molecular subtypes (**Figure 5C**).

Next, we explored the role of the hypoxia risk score in predicting the therapeutic response for several treatment options. Patients in the high-risk score group showed that they might be more sensitive to chemotherapeutic drugs, including Cisplatin, Docetaxel, Paclitaxel, Bleomycin, Camptothecin, and Vinblastine (**Figures 5D, E** **)**. In addition, the enrichment scores of several immunosuppressive oncogenic pathways were significantly higher in the low-risk score group, such as the WNT-β-catenin network, PPARG network, and FGFR3 network. As a result, blocking these oncogenic pathways may benefit patients in the low-risk score group (**Figure 5F**). Conversely, the enrichment scores of radiotherapy and EGFR-targeted therapy signatures were significantly higher in the high-risk score group. Therefore, patients in the high-risk score group may be more sensitive to radiotherapy and EGFR-targeted therapy (**Figure 5F**).

### Validating the Roles of the Hypoxia Risk Score in the Xiangya Cohort, GSE13507, and GSE32894

**Figure 6:**
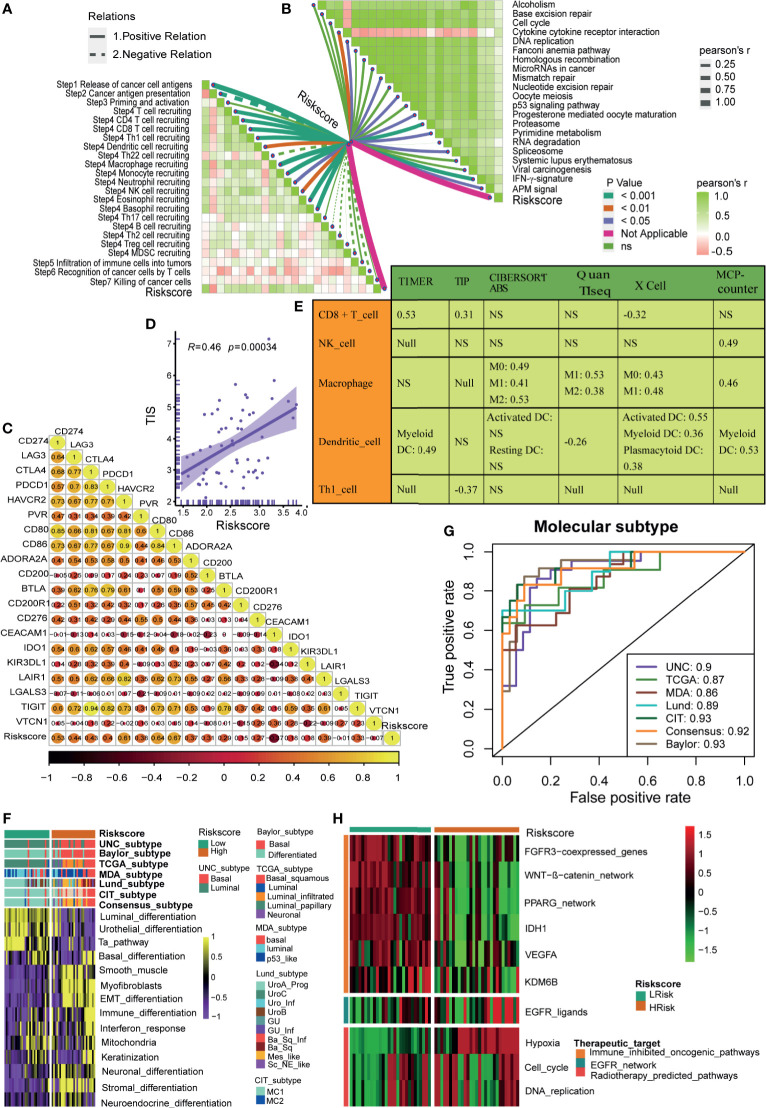
Roles of hypoxia risk score in the Xiangya cohort. **(A)** Correlations between hypoxia risk score and the activities of the cancer immunity cycles. **(B)** Correlations between hypoxia risk score and the enrichment scores of immunotherapy-predicted pathways. **(C)** Correlations between hypoxia risk score and immune checkpoints. **(D)** Correlations between hypoxia ris score and the T cell inflamed score (TIS). **(E)** Correlations between hypoxia risk score and the infiltration levels of five tumor infiltrating immune cells (CD8+ T cells, NK cells, macrophages, Th1 cells, and dendritic cells). **(F)** The associations between the hypoxia risk score groups and the molecular subtypes in seven different algorithms. **(G)** The predictive accuracy of hypoxia risk score for molecular subtypes in seven different algorithms. **(H)** Correlations between hypoxia risk score and the enrichment scores of several therapeutic signatures such as targeted therapies and radiotherapy.

In our cohort (Xiangya cohort), we further validated the role of the hypoxia risk score in predicting the immune phenotypes, molecular subtypes, and therapeutic opportunities. The hypoxia risk score was positively related to the enrichment scores of anti-cancer immunity cycles (**Figure 6A**). Consistently, the hypoxia risk score was positively related to the infiltration levels of several corresponding TIICs, such as the CD8 T cells, NK cells, and Dendritic cells (**Figure 6E**). Meanwhile, the hypoxia risk score was also positively related to immune checkpoints, TIS, and enrichment scores of ICB response-related signatures (**Figures 6B–D**). Therefore, the high-risk score group also indicated an inflamed phenotype in the Xiangya cohort. Furthermore, the hypoxia risk score could accurately stratify the molecular subtypes and corresponding subtypes specific signatures in the Xiangya cohort (**Figure 6F**). The AUC ranged from 0.86 to 0.93 in seven independent systems (**Figure 6G**). As expected, the hypoxia risk score could predict the clinical response of radiotherapy and several targeted therapies (**Figure 6H**). Patients in the high-risk score group may be more sensitive to radiotherapy and EGFR targeted therapy. However, targeted therapies, such as blocking the FGFR3 network and blocking the WNT-β-catenin network, may be more suitable for patients in the low-risk score group. All the above results were successfully validated in GSE13507 and GSE32894 (**Supplementary Figures 12** and **13**).

## Discussion

Many hypoxia risk scores have been developed to predict the cancer prognosis and TME characters (8–11). But there is no research to systematically correlate the hypoxia-related signature with the TME characters in BLCA. Here, we developed and validated a novel hypoxia risk score by integrating multiple independent BLCA data sets and the Xiangya cohort. The hypoxia risk score could predict the clinical outcomes, molecular subtypes, and TME characteristics. In addition, the hypoxia risk score may predict the efficacy of the ICB, chemotherapy, radiotherapy, and targeted therapies in BLCA.

Hypoxia is a common feature in TME of various cancers (3, 5, 23). Tumor cells undergo metabolic reprogramming, especially glucose metabolism, to adapt to the hypoxic TME (24–26). Consistently, we found that the hypoxia DEGs were significantly enriched in glucose metabolism-related pathways (**Supplementary Figures 1C, D**). The hypoxia risk score could reflect the actual hypoxia states in TME from several aspects. First, the hypoxia risk score could predict the prognosis and clinical characters of BLCA. A higher risk score indicated poorer OS, advanced tumor grade and stage. Second, we analyzed the differences in the enrichment scores of 50 hallmark pathways between the hypoxia risk score groups. As expected, hypoxia, angiogenesis, and glycolysis pathways were significantly enriched in the high-risk score group. In addition, other cancer-associated pathways, such as the P53 pathway, NOTCH signaling, DNA repair signaling, and KARS signaling, were also enriched in the high-risk score group. Chemotherapy was the most important treatment for advanced BLCA (2). It is necessary to develop accurate predictors of chemotherapy sensitivity to pinpoint the best candidates to receive chemotherapy. Here, we found that the sensitivities of the six most commonly used chemotherapeutic drugs in BLCA were significantly higher in the high-risk score group; this suggested that patients with high-risk score may benefit more from chemotherapy.

Yang et al. developed a 24-gene hypoxia signature in BLCA (12). They found that patients with high-risk score had a worse prognosis. Meanwhile, they demonstrated that the hypoxia risk score aided in selecting patients likely to benefit from the addition of carbogen and nicotinamide to radiotherapy. Consistently, we found that patients in the high-risk score group had a poorer prognosis. In addition, patients in the high-risk score group may be more sensitive to radiotherapy. Nonetheless, there are several different focuses between our study and Yang’s research. First, the selected hypoxia gene set was different between the two studies. Yang et al. derived their hypoxia signature based on 611 hypoxia-regulated genes from previously published literature (27). In our study, we developed our hypoxia risk score based on the hallmark hypoxia signature which included 200 genes that are up-regulated in response to low oxygen levels (hypoxia). Compared to other published hypoxia gene sets, the hallmark hypoxia signature reduced redundancy and produced a more robust enrichment analysis result (21). Second, Yang et al. calculated the hypoxia risk score by directly using the median expression of genes associated with poor prognosis. In our study, we generated the hypoxia risk score by integrating the differential expression analysis, Cox analysis, and LASSO algorithm. Third, Yang et al. did not analyze the association between the hypoxia risk score and TME characters, especially the immune characters. In our study, we comprehensively correlate the hypoxia risk score with several TME immune features, such as the TIICs, immune checkpoints, and TIS.

Hypoxia plays a critical role in regulating the tumor immune microenvironment *via* various mechanisms. Hypoxia upregulates the expression of several inhibitory immune molecules to shape an immunosuppressive TME. For instance, *via* HIF-1, hypoxia directly upregulates the expression of PD-L1 in various tumor cells by directly binding the HRE in the promoter of the PD-L1 gene (28). Hypoxia also promotes the immunosuppressive function of MDSC by upregulating the VISTA expression (29), induces tumor cell escape from phagocytosis by upregulating the CD47 (30), and stimulates the expression of the Non-Classical MHC class I (HLA-G) to inhibit the function of several immune cells, including B cells, T cells, natural killer cells, and dendritic cells (31, 32). Under a hypoxic TME, cancer cells continue ATP production by switching to glycolysis, which leads to the accumulation of immunosuppressive lactic acid and adenosine (33). Low pH condition, caused by the accumulation of excessive lactic acid, inhibits the secretion of IL-2, tumor necrosis factors, and IFN-γ from T lymphocytes (34, 35). So, CD8+ T cells’ cytotoxic activity was also markedly inhibited under such a low pH condition (36). The adenosine accumulated in the TME negatively regulates the activation of the anti-tumor T cell response (37–39). In addition, many other mechanisms have also been explored. Hypoxia activates autophagy to degrade the proapoptotic protein GZMB, thus inhibiting NK-mediated killing of cancer cells (40). Hypoxia upregulates the infiltration levels of Treg cells by increasing the expression of the FOXP3 transcription factor, TGF-β, and CCL28, which may inhibit the anti-cancer immune responses (41, 42). Overall, these data prompt us to explore the role of the hypoxia risk score in predicting the immune characters in TME.

In this study, the hypoxia risk score was positively correlated with the TIS, the enrichment scores of anti-cancer immunity cycles (such as T cell recruiting and release of cancer cell antigen), and TIICs (such as CD8 T cells and NK cells), which suggested that there was a higher pre-existing anti-cancer immunity in the TME of patients in the high-risk score group (43). However, this pre-existing anti-cancer immunity may be in a restrained state. That was because the hypoxia risk score was also positively correlated with M2 macrophages (**Figure 4B**), which was recognized as a cancer-promoting immune cell to inhibit the anti-cancer immunity, and its infiltration was positively regulated by hypoxia (44, 45). As we all know, immune checkpoints inhibit the anti-cancer immunity in TME (46). Consistently, in this study, the hypoxia risk score was indeed positively correlated with the expression of many immune checkpoints, such as PD-L1, PD-1, and CTLA-4. Therefore, for patients in the high-risk score group, although the pre-existing anti-cancer immunity in TME was higher, it was suppressed by the higher infiltration level of M2 macrophages and the higher expression of immune checkpoints. So, patients in the high-risk score group may benefit more from treatments that can reactivate the suppressed anti-cancer immunity in TME, such as ICB (13). However, for patients in the low-risk score group, the TIS and the expression of immune checkpoints were significantly lower, which indicated lower anti-cancer immunity and fewer immunotherapy targets in TME. Therefore, patients in the low-risk score group may not be suitable for ICB.

There were a few limitations to this study. First, this study was performed by using bioinformatic analyses. Though we validated the results in our own cohort and several public cohorts, we did not explore the relevant mechanisms of hypoxia *in vivo* or *in vitro*. Second, the clinical value of our hypoxia risk score needs further validation in prospective clinical trials. Third, we did not determine the optimal cut-off value of the hypoxia risk score. Alternatively, the median of the hypoxia risk score was defined as the cut-off value in all the validation cohorts.

In conclusion, we developed and validated a novel hypoxia risk score, which could predict the clinical outcomes and the TME characteristics of BLCA. The hypoxia risk score may aid in the development of precision medicine in BLCA. For patients in the high-risk score group, they may benefit from immunotherapy, chemotherapy, radiotherapy, and EGFR targeted therapy. In contrast, patients in the low-risk score group may benefit from several targeted therapies, such as blocking the WNT-β-catenin network, PPARG network, and FGFR3 network.

## Data Availability Statement

The datasets presented in this study can be found in online repositories. The names of the repository/repositories and accession number(s) can be found in the article/**Supplementary Material**.

## Ethics Statement

Written informed consent was obtained from the individual(s) for the publication of any potentially identifiable images or data included in this article.

## Author Contributions

Conception and design: ZL, QT, JH, and XZ. Provision of study materials or patients: ZL, TQ, and BO. Collection and assembly of data: ZL, JH, XZ, ZY, and JC. Data analysis and interpretation: ZL, JH, XZ, ZY, and JC. Manuscript writing: All authors. All authors contributed to the article and approved the submitted version.

## Funding

This work was supported by the grants from the Science and Technology Joint Fund Project in Guizhou Province [LH(2016)7386], Guizhou Provincial Education Department Youth Science and Technology talent Growth Project [KY(2018)172], and the National Natural Science Foundation of China [82070785, 81873626, 81902592].

## Conflict of Interest

The authors declare that the research was conducted in the absence of any commercial or financial relationships that could be construed as a potential conflict of interest.

## Publisher’s Note

All claims expressed in this article are solely those of the authors and do not necessarily represent those of their affiliated organizations, or those of the publisher, the editors and the reviewers. Any product that may be evaluated in this article, or claim that may be made by its manufacturer, is not guaranteed or endorsed by the publisher.

## Supplementary Material

Supplementary Figure 1 Identification of differentially expressed hypoxia genes and functional analysis. **(A)** The top 20 differentially expressed hypoxia genes between BLCA and normal tissues. **(B)** The volcano plot of the differentially expressed hypoxia genes. **(C, D)** Go and KEGG analysis of the differentially expressed hypoxia genes. **(E)** The PPI network of the differentially expressed hypoxia genes.

Supplementary Figure 2 Subgroup survival analyses based on grade, stage, gender, and age in TCGA-BLCA cohort. **(A, B)** The K-M curves and ROC curves of hypoxia risk score in the high grade and low grade subgroups. **(C, D)** The K-M curves and ROC curves of hypoxia risk score in the high stage and low stage subgroups. **(E, F)** The K-M curves and ROC curves of hypoxia risk score in the female and male subgroups. **(G, H)** The K-M curves and ROC curves of hypoxia risk score in the younger and older subgroups.

Supplementary Figure 3 The associations between the hypoxia risk score and clinicopathological characters in three external cohorts. **(A–C)** Associations between the hypoxia risk score and tumor grade, stage, and recurrence in GSE13507. **(D)** Association between the hypoxia risk score and tumor grade in GSE32894. **(E, F)** Associations between the hypoxia risk score and tumor grade and stage in Xiangya cohort.

Supplementary Figure 4 The multivariate Cox analysis in three external cohorts. **(A)** The results of multivariate Cox analysis in GSE13507; **(B)** The results of multivariate Cox analysis in GSE32894; **(C)** The results of multivariate Cox analysis in Xiangya cohort.

Supplementary Figure 5 The ROC cureves and calibration curves of the nomogram in three external cohorts. **(A)** The ROC curves and calibration curves of the nomogram in GSE13507; **(B)** The ROC curves and calibration curves of the nomogram in GSE32894; **(C)** The ROC curves and calibration curves of the nomogram in Xiangya cohort.

Supplementary Figure 6 Hypoxia risk score correlated with immune characters of TME and predicted the clinical response of ICB in the high stage subgroup in TCGA-BLCA. **(A)** Differences in activities of the cancer immunity cycles between high- and low-risk score groups. **(B)** The correlations between the hypoxia risk score and several immune cells. **(C)** The correlations between the hypoxia risk score and T cell inflamed score (TIS). **(D)** The correlations between hypoxia risk score and the enrichment scores of immunotherapy-predicted pathways. **(E)** The correlations between hypoxia risk score and immune checkpoints.

Supplementary Figure 7 Hypoxia risk score correlated with immune characters of TME and predicted the clinical response of ICB in the low stage subgroup in TCGA-BLCA. **(A)** Differences in activities of the cancer immunity cycles between high- and low-risk score groups. **(B)** The correlations between the hypoxia risk score and several immune cells. **(C)** The correlations between the hypoxia risk score and T cell inflamed score (TIS). **(D)** The correlations between hypoxia risk score and the enrichment scores of immunotherapy-predicted pathways. **(E)** The correlations between hypoxia risk score and immune checkpoints.

Supplementary Figure 8 Hypoxia risk score correlated with immune characters of TME and predicted the clinical response of ICB in the female subgroup in TCGA-BLCA. **(A)** Differences in activities of the cancer immunity cycles between high- and low-risk score groups. **(B)** The correlations between the hypoxia risk score and several immune cells. **(C)** The correlations between the hypoxia risk score and T cell inflamed score (TIS). **(D)** The correlations between hypoxia risk score and the enrichment scores of immunotherapy-predicted pathways. **(E)** The correlations between hypoxia risk score and immune checkpoints.

Supplementary Figure 9 Hypoxia risk score correlated with immune characters of TME and predicted the clinical response of ICB in the male subgroup in TCGA-BLCA. **(A)** Differences in activities of the cancer immunity cycles between high- and low-risk score groups. **(B)** The correlations between the hypoxia risk score and several immune cells. **(C)** The correlations between the hypoxia risk score and T cell inflamed score (TIS). **(D)** The correlations between hypoxia risk score and the enrichment scores of immunotherapy-predicted pathways. **(E)** The correlations between hypoxia risk score and immune checkpoints.

Supplementary Figure 10 Hypoxia risk score correlated with immune characters of TME and predicted the clinical response of ICB in the older subgroup in TCGA-BLCA. **(A)** Differences in activities of the cancer immunity cycles between high- and low-risk score groups. **(B)** The correlations between the hypoxia risk score and several immune cells. **(C)** The correlations between the hypoxia risk score and T cell inflamed score (TIS). **(D)** The correlations between hypoxia risk score and the enrichment scores of immunotherapy-predicted pathways. **(E)** The correlations between hypoxia risk score and immune checkpoints.

Supplementary Figure 11 Hypoxia risk score correlated with immune characters of TME and predicted the clinical response of ICB in the younger subgroup in TCGA-BLCA. **(A)** Differences in activities of the cancer immunity cycles between high- and low-risk score groups. **(B)** The correlations between the hypoxia risk score and several immune cells. **(C)** The correlations between the hypoxia risk score and T cell inflamed score (TIS). **(D)** The correlations between hypoxia risk score and the enrichment scores of immunotherapy-predicted pathways. **(E)** The correlations between hypoxia risk score and immune checkpoints.

Supplementary Figure 12 Roles of hypoxia risk score in the GSE13507. **(A)** Correlations between hypoxia risk score and the activities of the cancer immunity cycles. **(B)** Correlations between hypoxia risk score and the enrichment scores of immunotherapy-predicted pathways. **(C)** Correlations between hypoxia risk score and immune checkpoints. **(D)** Correlations between hypoxia risk score and genes of the T cell inflamed score algorithm. **(E)** The associations between the hypoxia risk score groups and the molecular subtypes in seven different algorithms. **(F)** The predictive accuracy of hypoxia risk score for molecular subtypes in seven different algorithms. **(G)** Correlations between hypoxia risk score and the enrichment scores of several therapeutic signatures such as targeted therapies and radiotherapy.

Supplementary Figure 13 Roles of hypoxia risk score in the GSE32894. **(A)** Correlations between hypoxia risk score and the activities of the cancer immunity cycles. **(B)** Correlations between hypoxia risk score and the enrichment scores of immunotherapy-predicted pathways. **(C)** Correlations between hypoxia risk score and immune checkpoints. **(D)** Correlations between hypoxia ris score and genes of the T cell inflamed score algorithm. **(E)** The associations between the hypoxia risk score groups and the molecular subtypes in seven different algorithms. **(F)** The predictive accuracy of hypoxia risk score for molecular subtypes in seven different algorithms. **(G)** Correlations between hypoxia risk score and the enrichment scores of several therapeutic signatures such as targeted therapies and radiotherapy.
