# Supplementary figures and images for: A Robust Hypoxia Risk Score Predicts the Clinical Outcomes and Tumor Microenvironment Immune Characters in Bladder Cancer
Source: Front Immunol. 2021 Aug 13;12:725223. doi: 10.3389/fimmu.2021.725223 (PMC8415032; doi:10.3389/fimmu.2021.725223)

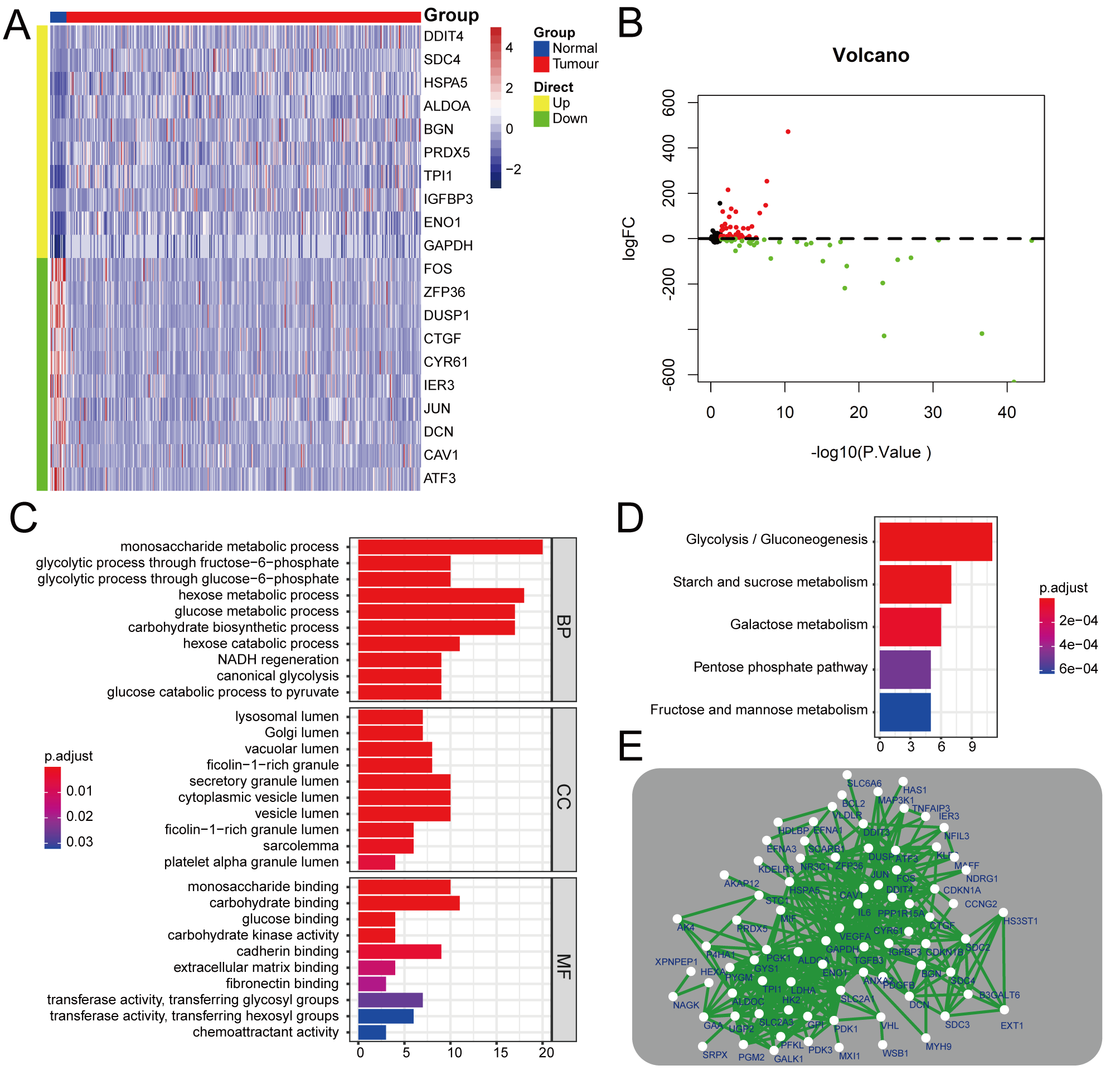

Supplement: Supplementary Figure 1 — Identification of differentially expressed hypoxia genes and functional analysis. (A) The top 20 differentially expressed hypoxia genes between BLCA and normal tissues. (B) The volcano plot of the differentially expressed hypoxia genes. (C, D) Go and KEGG analysis of the differentially expressed hypoxia genes. (E) The PPI network of the differentially expressed hypoxia genes. [file Image_1.tif]

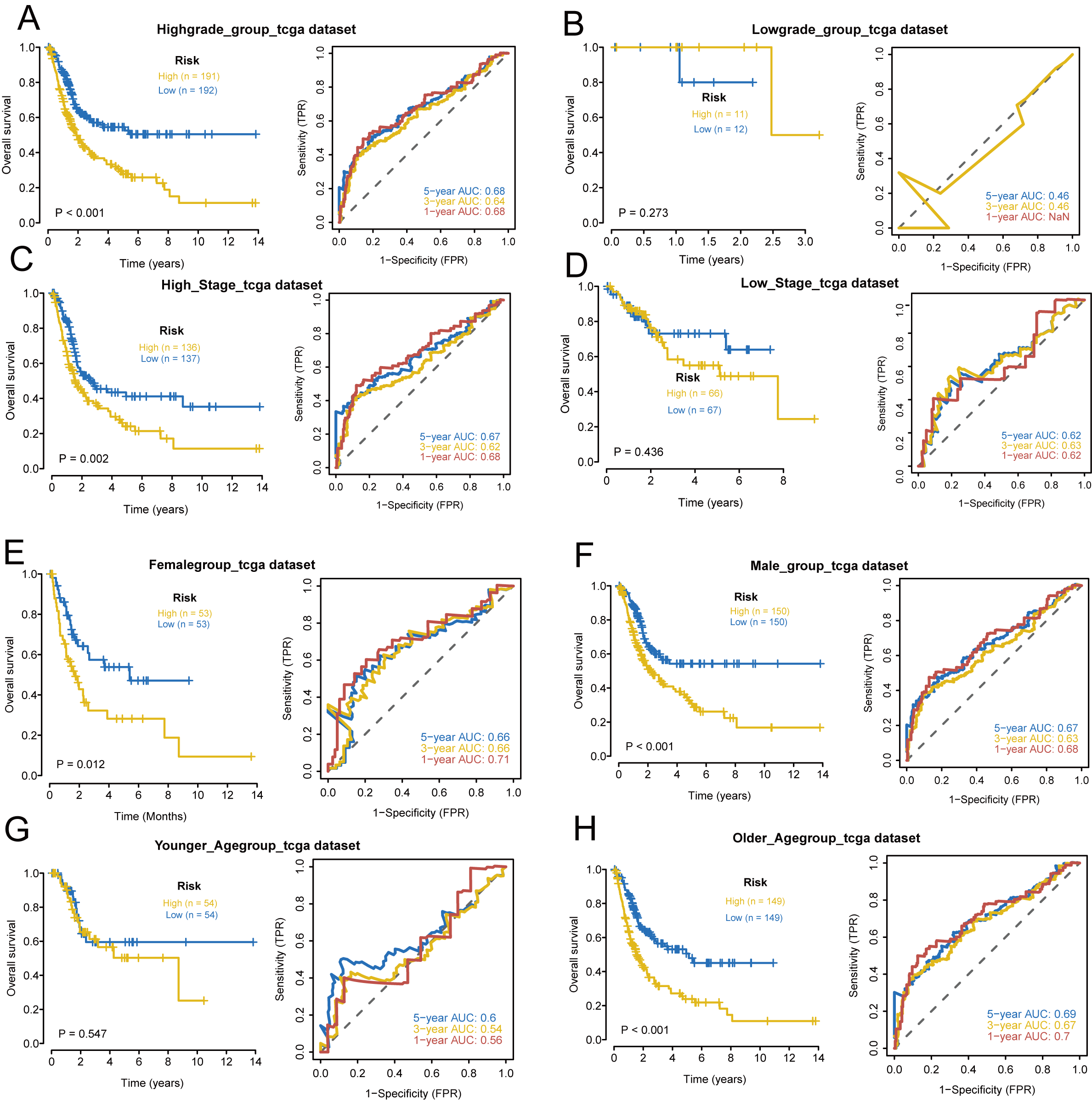

Supplement: Supplementary Figure 2 — Subgroup survival analyses based on grade, stage, gender, and age in TCGA-BLCA cohort. (A, B) The K-M curves and ROC curves of hypoxia risk score in the high grade and low grade subgroups. (C, D) The K-M curves and ROC curves of hypoxia risk score in the high stage and low stage subgroups. (E, F) The K-M curves and ROC curves of hypoxia risk score in the female and male subgroups. (G, H) The K-M curves and ROC curves of hypoxia risk score in the younger and older subgroups. [file Image_2.tif]

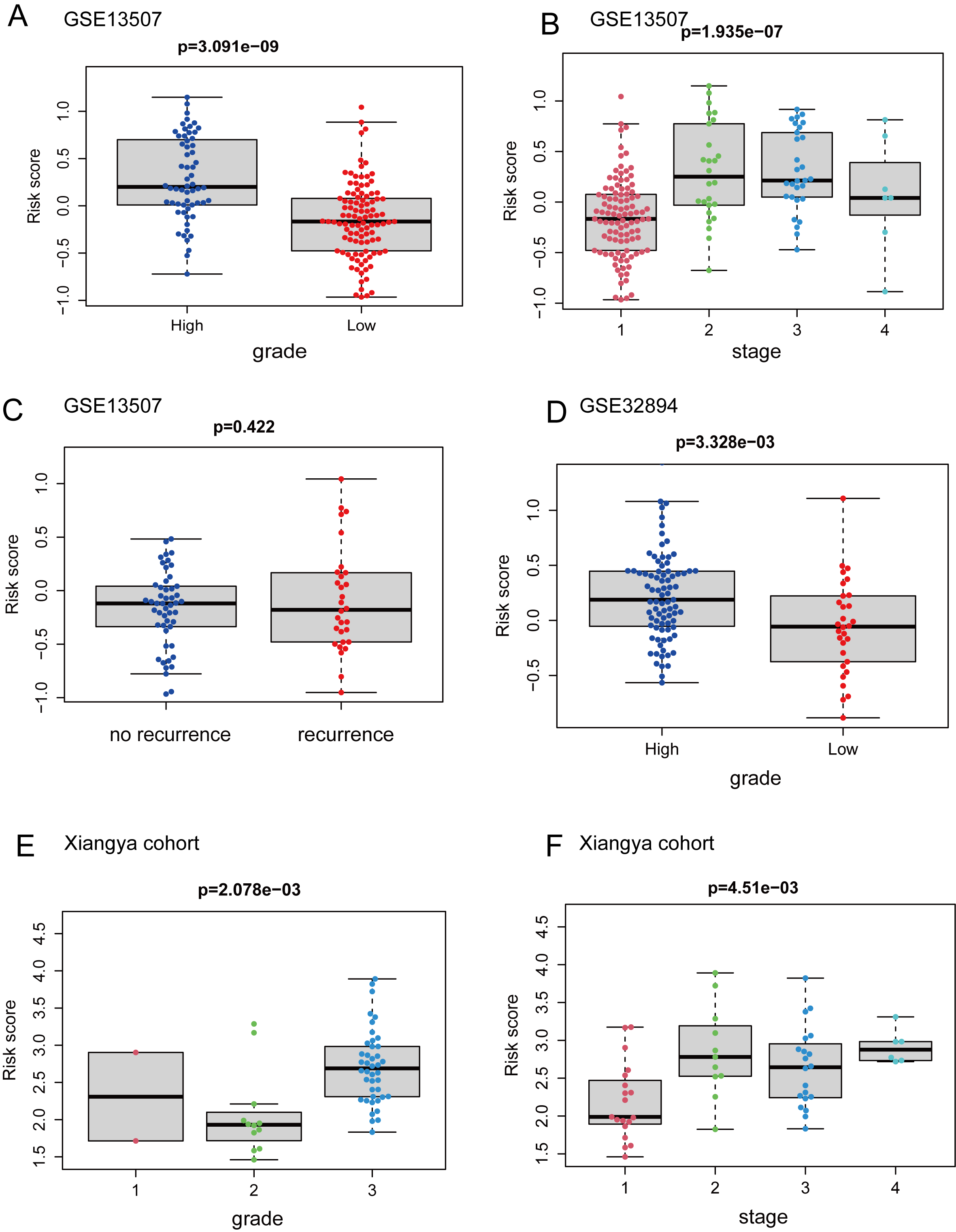

Supplement: Supplementary Figure 3 — The associations between the hypoxia risk score and clinicopathological characters in three external cohorts. (A–C) Associations between the hypoxia risk score and tumor grade, stage, and recurrence in GSE13507. (D) Association between the hypoxia risk score and tumor grade in GSE32894. (E, F) Associations between the hypoxia risk score and tumor grade and stage in Xiangya cohort. [file Image_3.tif]

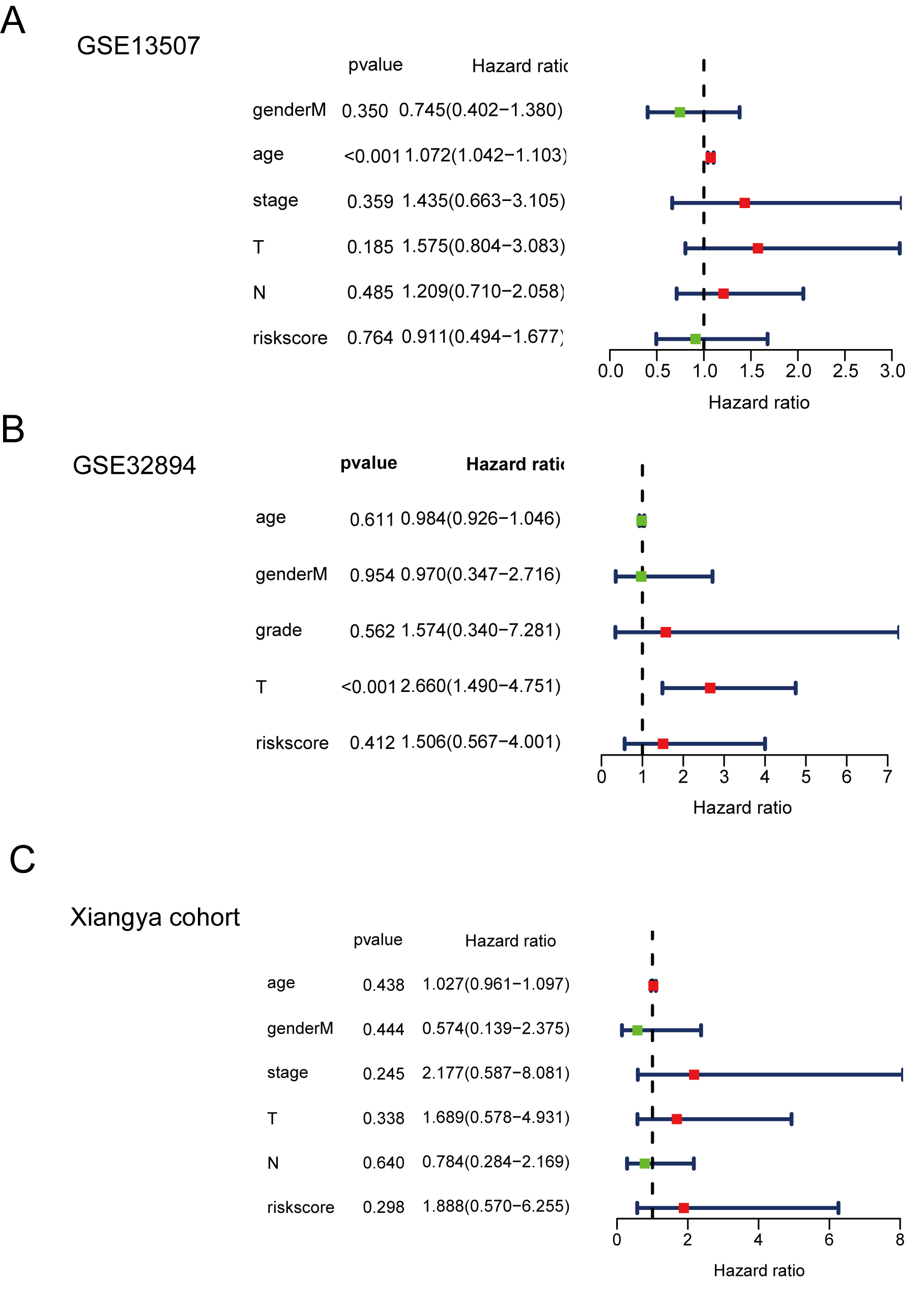

Supplement: Supplementary Figure 4 — The multivariate Cox analysis in three external cohorts. (A) The results of multivariate Cox analysis in GSE13507; (B) The results of multivariate Cox analysis in GSE32894; (C) The results of multivariate Cox analysis in Xiangya cohort. [file Image_4.tif]

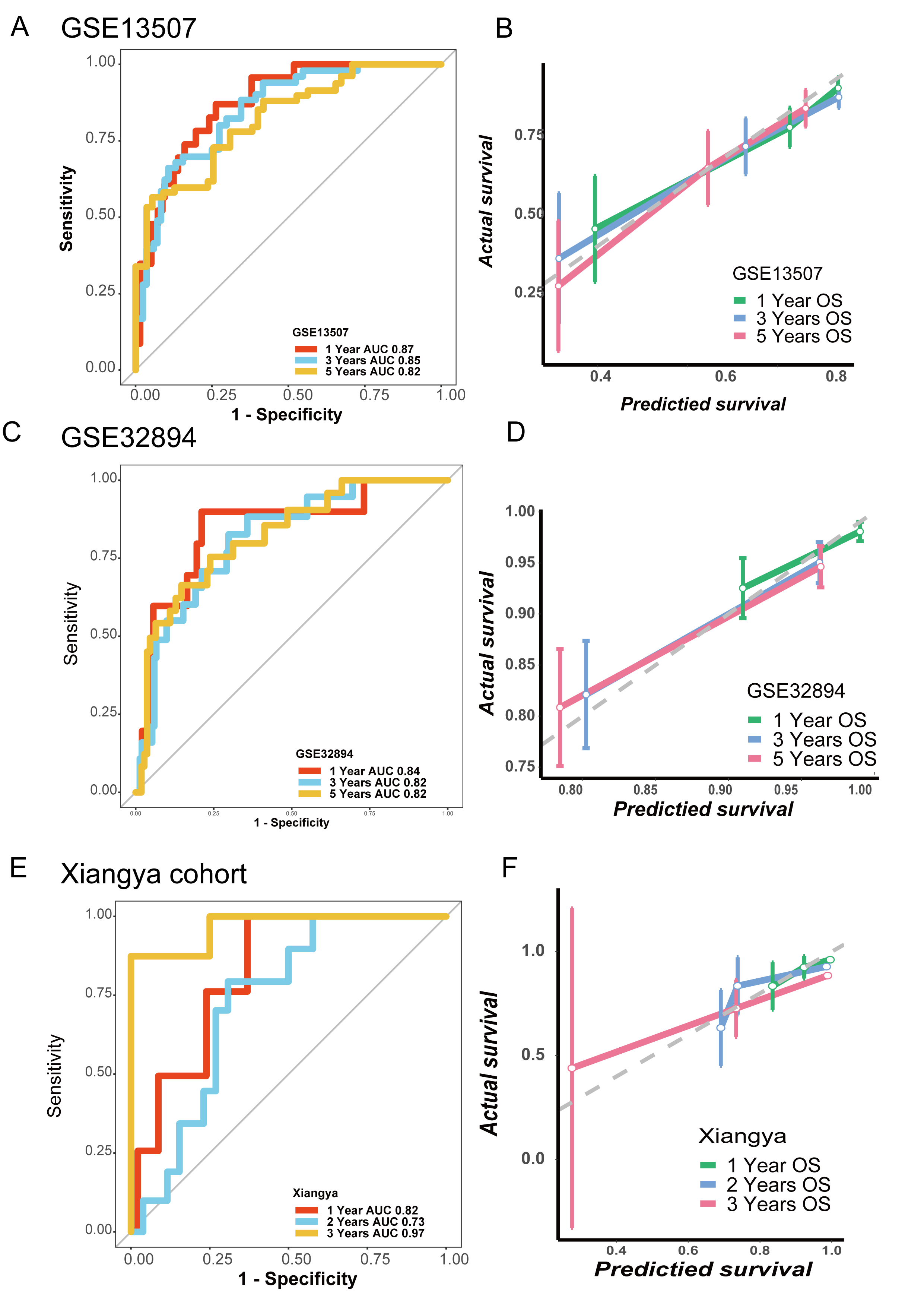

Supplement: Supplementary Figure 5 — The ROC cureves and calibration curves of the nomogram in three external cohorts. (A) The ROC curves and calibration curves of the nomogram in GSE13507; (B) The ROC curves and calibration curves of the nomogram in GSE32894; (C) The ROC curves and calibration curves of the nomogram in Xiangya cohort. [file Image_5.tif]

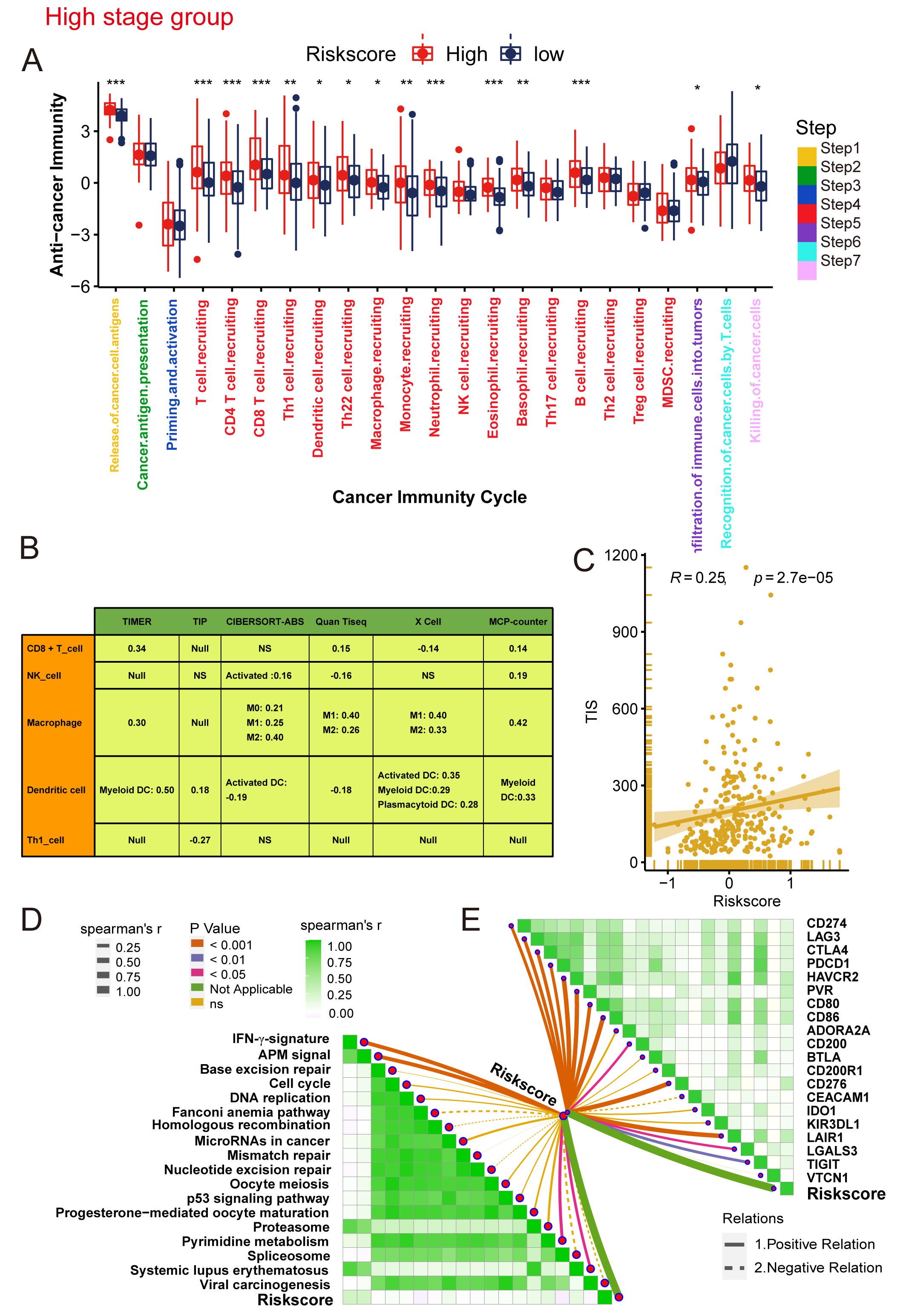

Supplement: Supplementary Figure 6 — Hypoxia risk score correlated with immune characters of TME and predicted the clinical response of ICB in the high stage subgroup in TCGA-BLCA. (A) Differences in activities of the cancer immunity cycles between high- and low-risk score groups. (B) The correlations between the hypoxia risk score and several immune cells. (C) The correlations between the hypoxia risk score and T cell inflamed score (TIS). (D) The correlations between hypoxia risk score and the enrichment scores of immunotherapy-predicted pathways. (E) The correlations between hypoxia risk score and immune checkpoints. [file Image_6.tif]

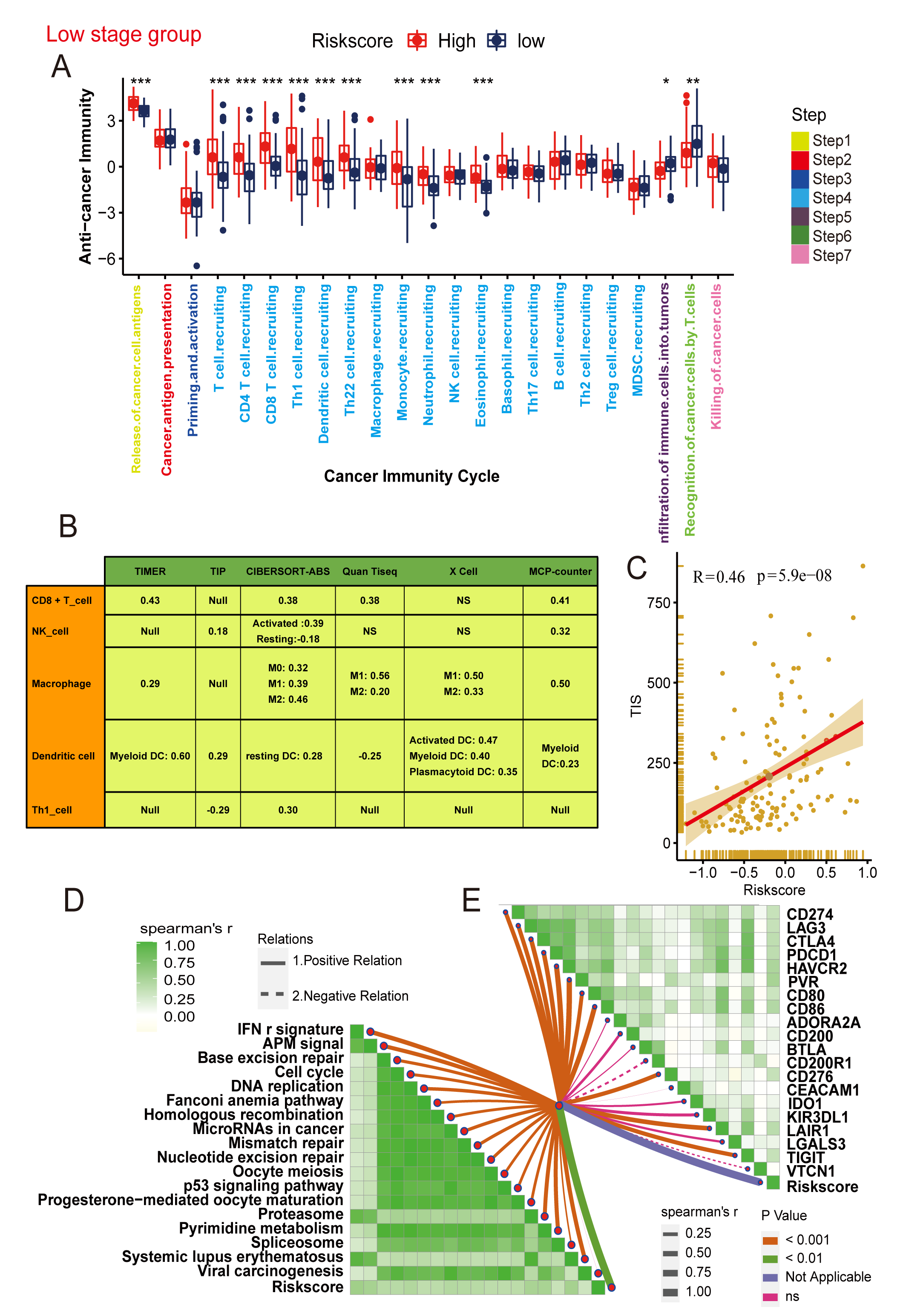

Supplement: Supplementary Figure 7 — Hypoxia risk score correlated with immune characters of TME and predicted the clinical response of ICB in the low stage subgroup in TCGA-BLCA. (A) Differences in activities of the cancer immunity cycles between high- and low-risk score groups. (B) The correlations between the hypoxia risk score and several immune cells. (C) The correlations between the hypoxia risk score and T cell inflamed score (TIS). (D) The correlations between hypoxia risk score and the enrichment scores of immunotherapy-predicted pathways. (E) The correlations between hypoxia risk score and immune checkpoints. [file Image_7.tif]

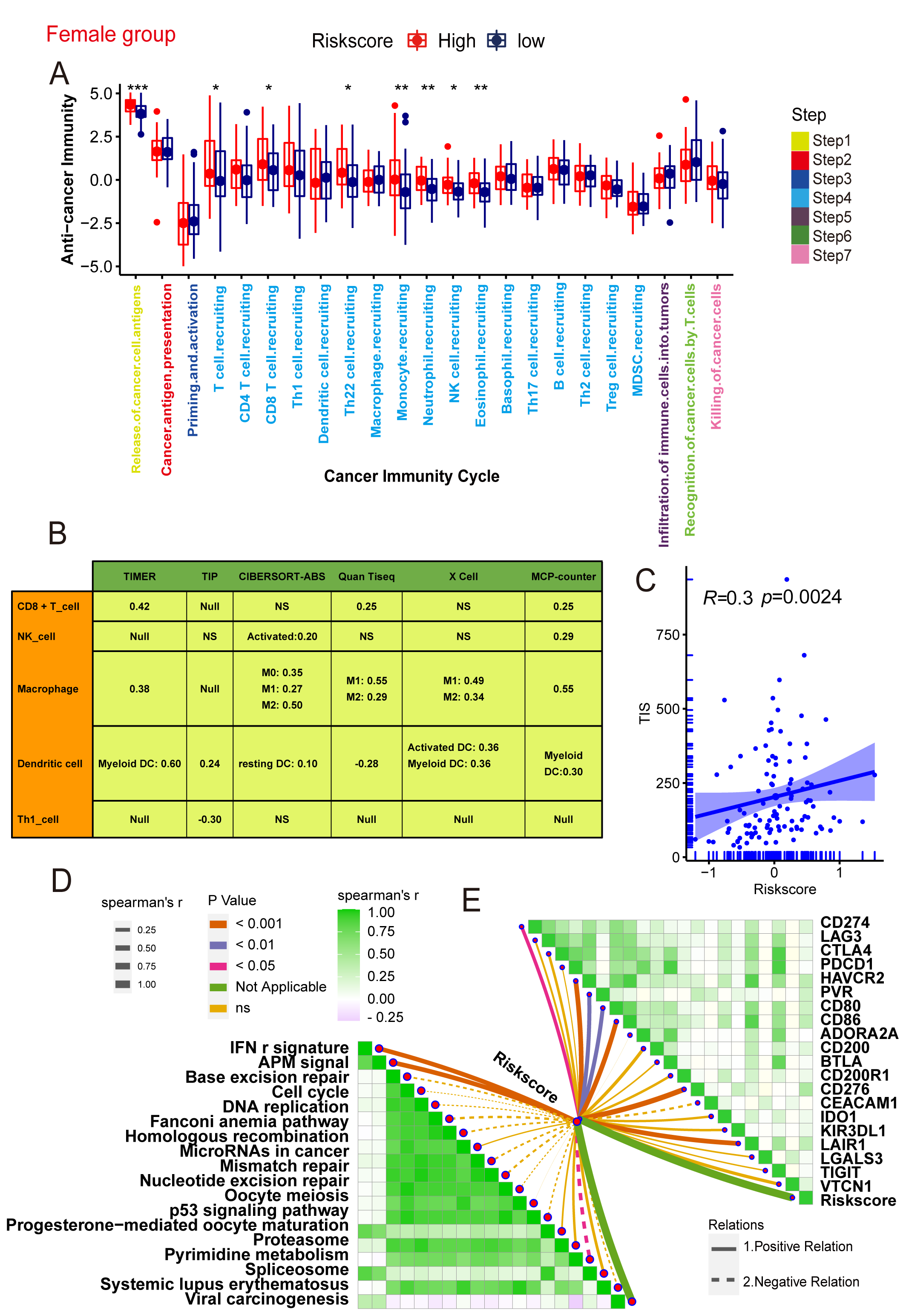

Supplement: Supplementary Figure 8 — Hypoxia risk score correlated with immune characters of TME and predicted the clinical response of ICB in the female subgroup in TCGA-BLCA. (A) Differences in activities of the cancer immunity cycles between high- and low-risk score groups. (B) The correlations between the hypoxia risk score and several immune cells. (C) The correlations between the hypoxia risk score and T cell inflamed score (TIS). (D) The correlations between hypoxia risk score and the enrichment scores of immunotherapy-predicted pathways. (E) The correlations between hypoxia risk score and immune checkpoints. [file Image_8.tif]

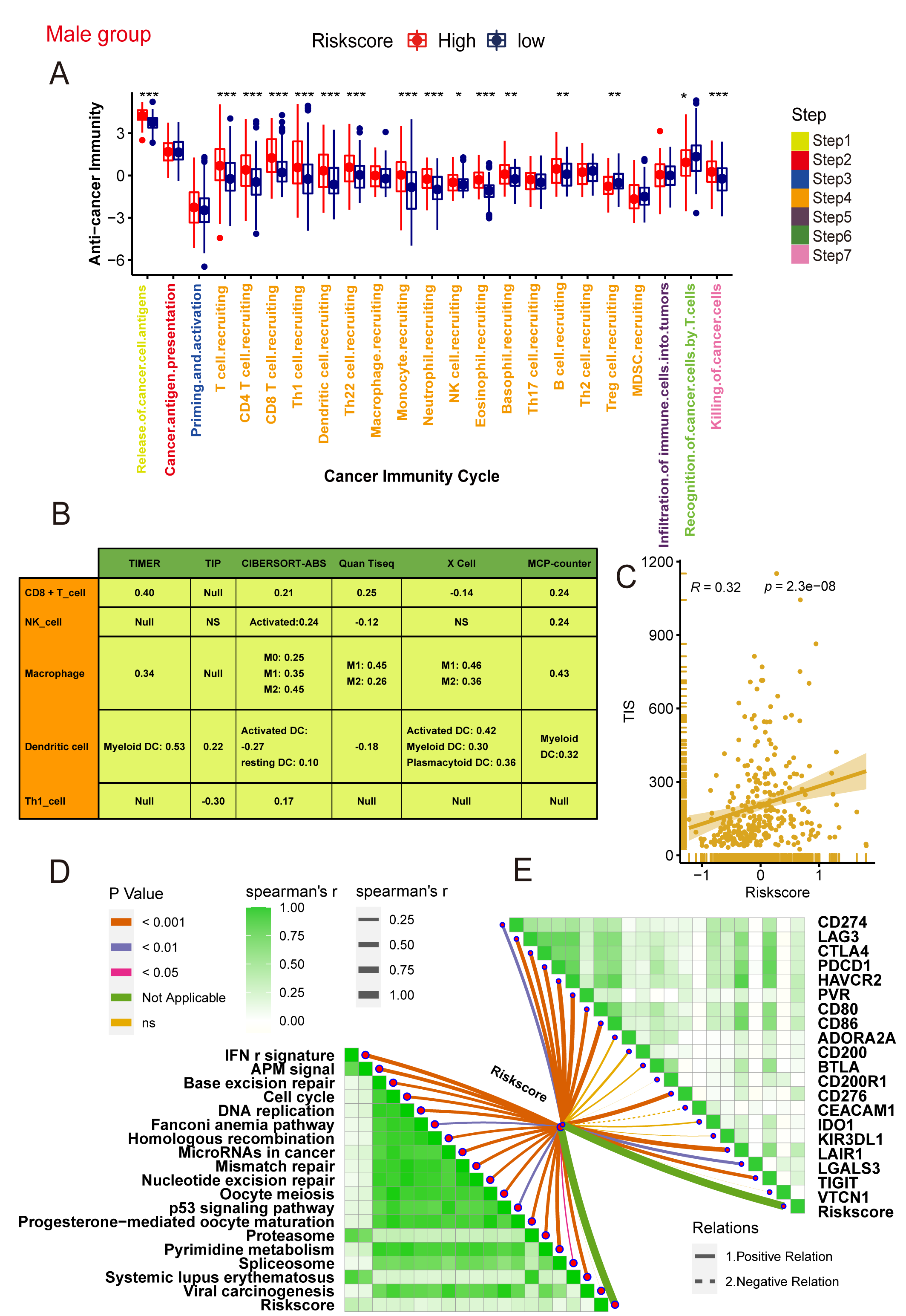

Supplement: Supplementary Figure 9 — Hypoxia risk score correlated with immune characters of TME and predicted the clinical response of ICB in the male subgroup in TCGA-BLCA. (A) Differences in activities of the cancer immunity cycles between high- and low-risk score groups. (B) The correlations between the hypoxia risk score and several immune cells. (C) The correlations between the hypoxia risk score and T cell inflamed score (TIS). (D) The correlations between hypoxia risk score and the enrichment scores of immunotherapy-predicted pathways. (E) The correlations between hypoxia risk score and immune checkpoints. [file Image_9.tif]

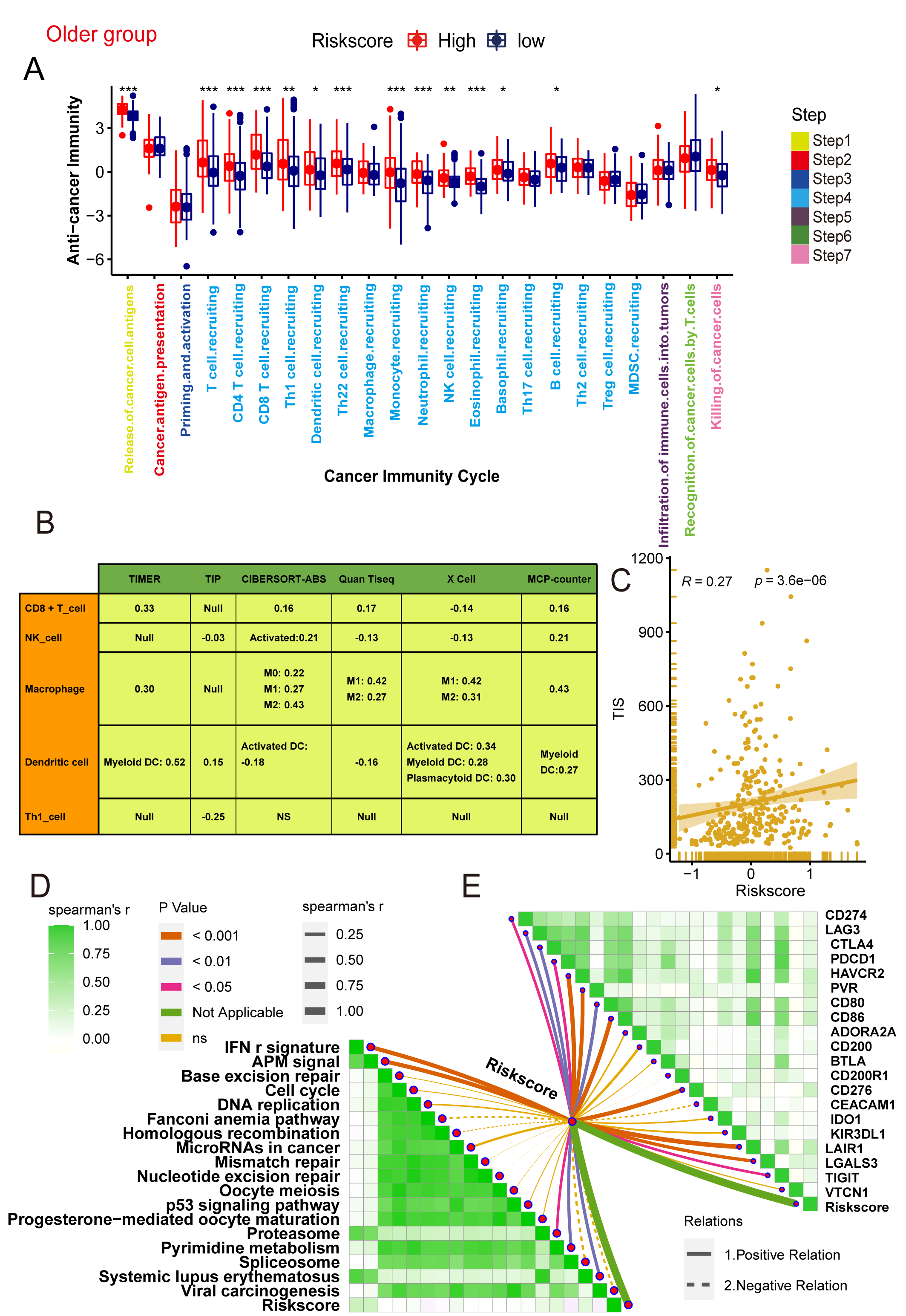

Supplement: Supplementary Figure 10 — Hypoxia risk score correlated with immune characters of TME and predicted the clinical response of ICB in the older subgroup in TCGA-BLCA. (A) Differences in activities of the cancer immunity cycles between high- and low-risk score groups. (B) The correlations between the hypoxia risk score and several immune cells. (C) The correlations between the hypoxia risk score and T cell inflamed score (TIS). (D) The correlations between hypoxia risk score and the enrichment scores of immunotherapy-predicted pathways. (E) The correlations between hypoxia risk score and immune checkpoints. [file Image_10.tif]

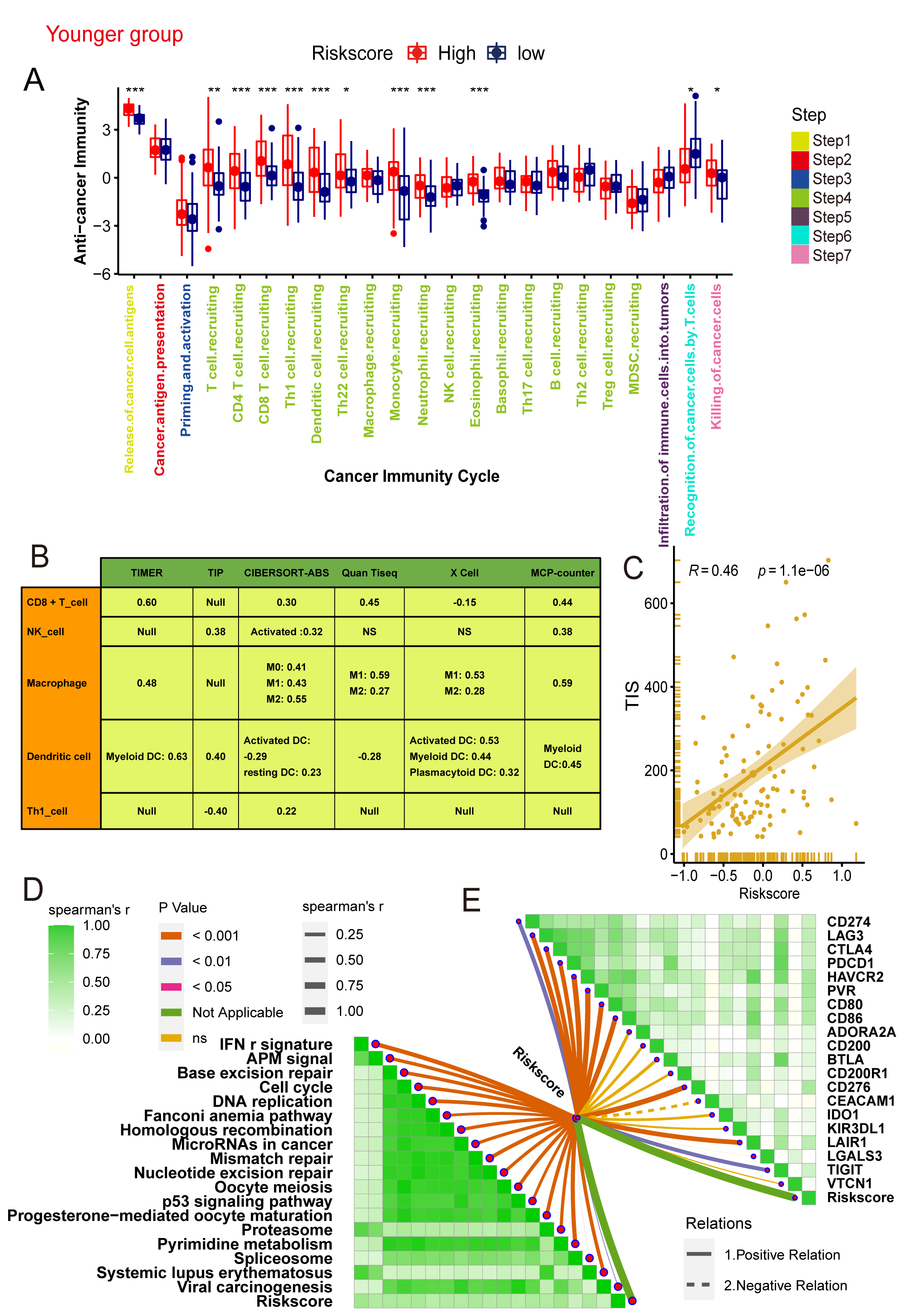

Supplement: Supplementary Figure 11 — Hypoxia risk score correlated with immune characters of TME and predicted the clinical response of ICB in the younger subgroup in TCGA-BLCA. (A) Differences in activities of the cancer immunity cycles between high- and low-risk score groups. (B) The correlations between the hypoxia risk score and several immune cells. (C) The correlations between the hypoxia risk score and T cell inflamed score (TIS). (D) The correlations between hypoxia risk score and the enrichment scores of immunotherapy-predicted pathways. (E) The correlations between hypoxia risk score and immune checkpoints. [file Image_11.tif]

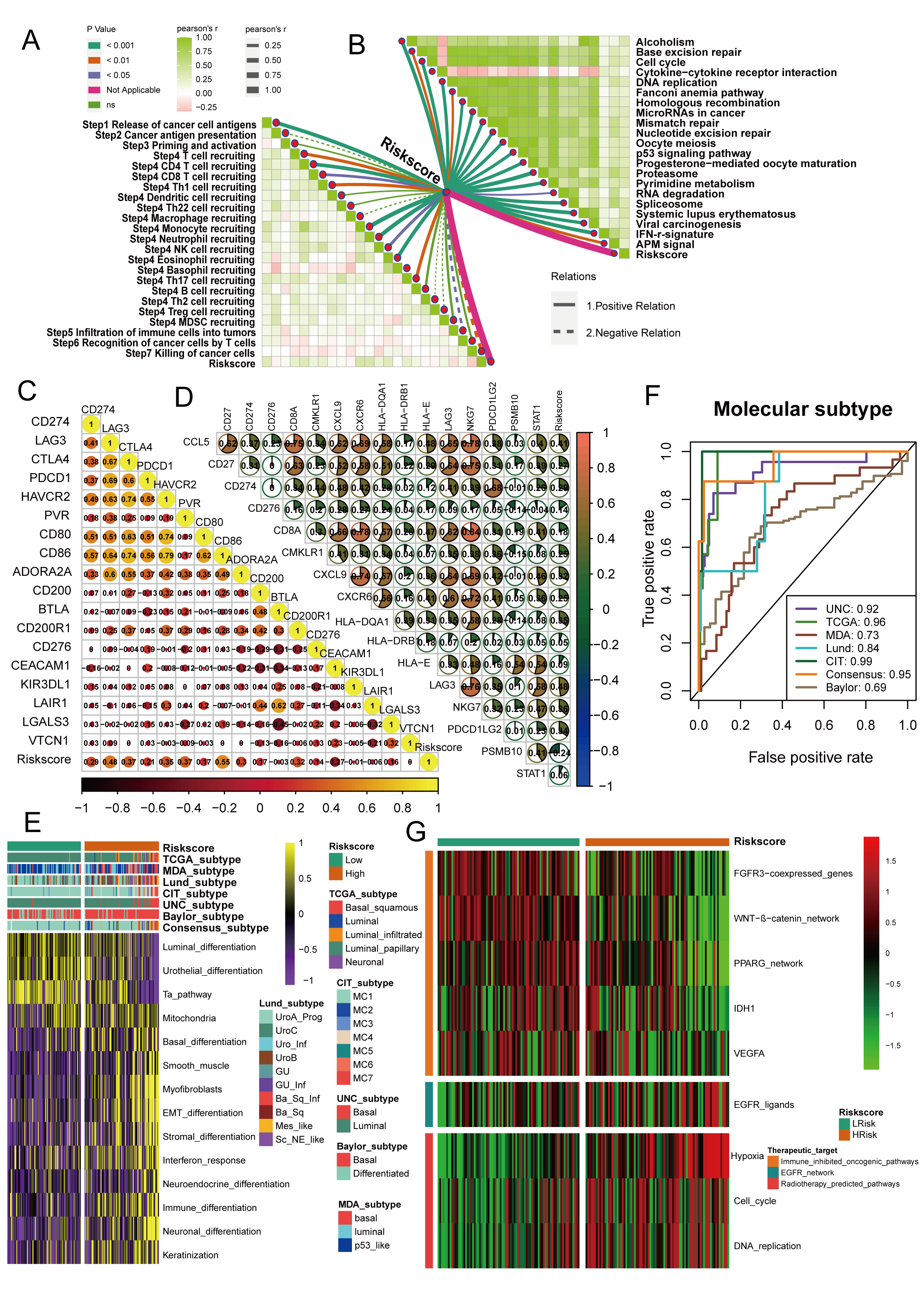

Supplement: Supplementary Figure 12 — Roles of hypoxia risk score in the GSE13507. (A) Correlations between hypoxia risk score and the activities of the cancer immunity cycles. (B) Correlations between hypoxia risk score and the enrichment scores of immunotherapy-predicted pathways. (C) Correlations between hypoxia risk score and immune checkpoints. (D) Correlations between hypoxia risk score and genes of the T cell inflamed score algorithm. (E) The associations between the hypoxia risk score groups and the molecular subtypes in seven different algorithms. (F) The predictive accuracy of hypoxia risk score for molecular subtypes in seven different algorithms. (G) Correlations between hypoxia risk score and the enrichment scores of several therapeutic signatures such as targeted therapies and radiotherapy. [file Image_12.tif]

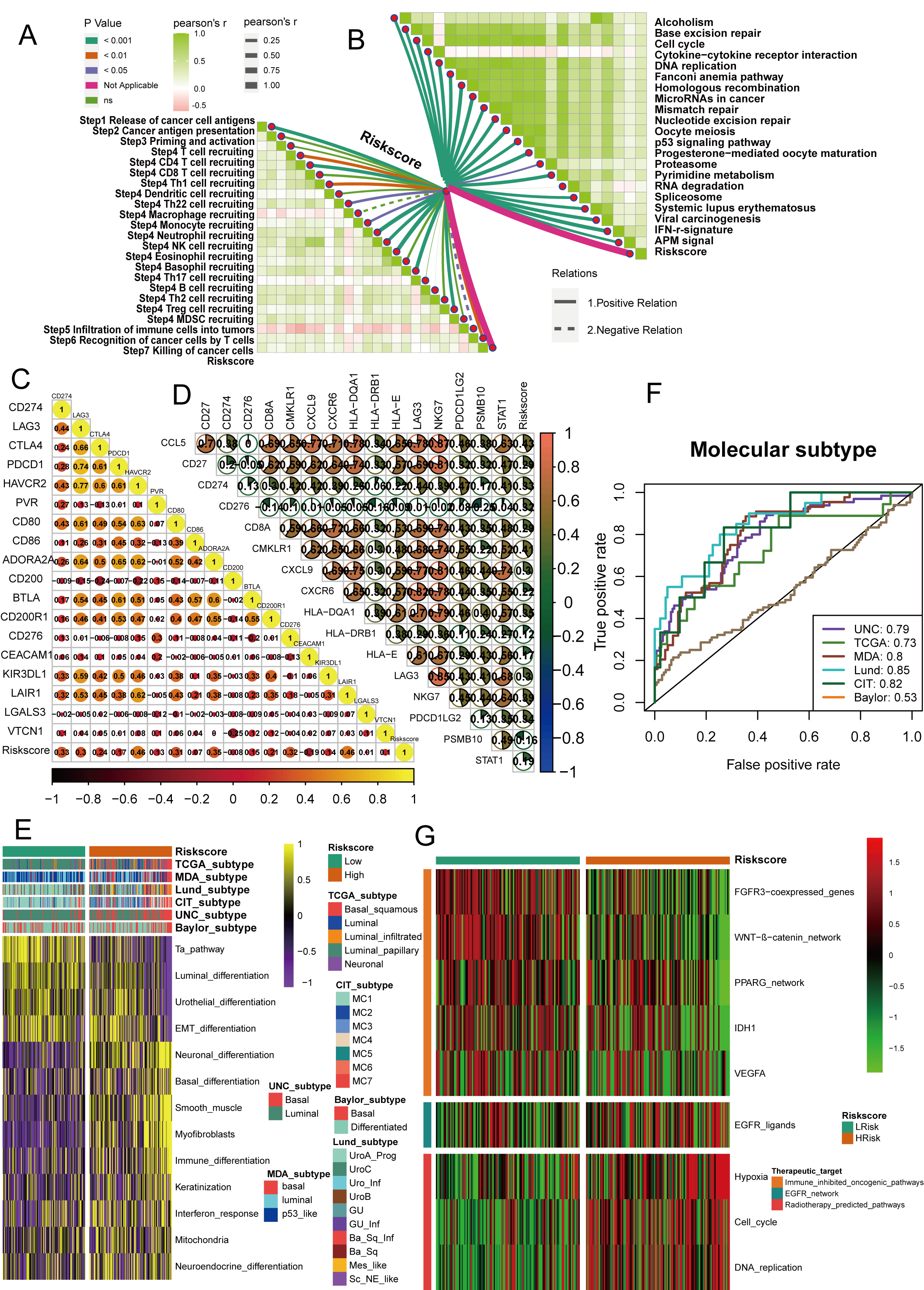

Supplement: Supplementary Figure 13 — Roles of hypoxia risk score in the GSE32894. (A) Correlations between hypoxia risk score and the activities of the cancer immunity cycles. (B) Correlations between hypoxia risk score and the enrichment scores of immunotherapy-predicted pathways. (C) Correlations between hypoxia risk score and immune checkpoints. (D) Correlations between hypoxia ris score and genes of the T cell inflamed score algorithm. (E) The associations between the hypoxia risk score groups and the molecular subtypes in seven different algorithms. (F) The predictive accuracy of hypoxia risk score for molecular subtypes in seven different algorithms. (G) Correlations between hypoxia risk score and the enrichment scores of several therapeutic signatures such as targeted therapies and radiotherapy. [file Image_13.tif]
